# PFAS exposure and reproductive dysfunction: implications for outcomes in assisted reproductive technologies

**DOI:** 10.3389/fendo.2026.1808266

**Published:** 2026-05-14

**Authors:** Xin Shu, Wen-Jie Zhou, Tao Zhang, Jiang-Feng Ye, Yan-Jun Zhao, Chun-Jie Gu, Ying-Ying Yu, Ming-Qing Li

**Affiliations:** 1 Department of Reproductive Immunology, The International Peace Maternity and Child Health Hospital, School of Medicine, Shanghai Jiao Tong University, Shanghai, China; 2 Shanghai Key Laboratory of Embryo Original Diseases, Shanghai, China; 3 Reproductive Medical Center, Department of Obstetrics and Gynecology, Ruijin Hospital, Shanghai Jiao Tong University School of Medicine, Shanghai, China; 4 Assisted Reproductive Technology Unit, Department of Obstetrics and Gynecology, Faculty of Medicine, Chinese University of Hong Kong, Hong Kong, Hong Kong SAR, China; 5 Institute for Molecular and Cell Biology, Agency for Science, Technology and Research, Singapore, Singapore; 6 Department of Child Health Care, Shanghai Children’s Hospital, School of Medicine, Shanghai Jiao Tong University, Shanghai, China

**Keywords:** environmental pollutants, fertility, PFAS, reproductive health, reproductive outcomes

## Abstract

The high stability and bioaccumulation potential of per- and polyfluoroalkyl substances (PFAS) have led to their widespread environmental presence and raised concerns about human health exposure through multiple pathways. In particular, PFAS exposure during assisted reproductive technology (ART) has attracted increasing attention from the scientific community. Current evidence indicates that PFAS may be associated with diminished fertility, adverse pregnancy outcomes, and impaired fetal health, although the underlying mechanisms remain incompletely elucidated. Emerging studies suggest that these effects may arise from multi-level disruptions of the reproductive system, including interference with hypothalamic–pituitary–gonadal axis regulation, steroidogenesis, gamete quality, oxidative stress and inflammatory signaling, as well as compromised endometrial receptivity and placental function. This review synthesizes and analyzes existing research to explore the impact of PFAS on reproductive outcomes following ART. It highlights potential risks to reproductive health and offers updated perspectives for researchers and public health policymakers.

## 1 Introduction

Per- and polyfluoroalkyl substances (PFASs) are a class of highly stable synthetic compounds widely used in industrial and consumer products, such as water-repellent coatings, cleaning agents, and food packaging materials. Due to the stability of their chemical structure, PFAS are extremely difficult to degrade in the environment, leading to widespread accumulation in water sources, soil, and living organisms (1). In recent years, accumulating evidence has demonstrated that exposure to PFAS is associated with multiple health issues, particularly concerning reproductive health. Assisted reproductive technology (ART), including *in vitro* fertilization (IVF) and intracytoplasmic sperm injection (ICSI), has become an essential clinical approach for the treatment of infertility, with its use increasing globally due to delayed childbearing and rising infertility rates (2, 3). Specifically, PFAS exposure has been suggested to be associated with changes in early ART-related outcomes, including oocyte quality and embryo development, whereas associations with clinical endpoints such as implantation and pregnancy outcomes remain less consistent. Because ART procedures involve hormonally regulated gametogenesis, embryo culture, implantation, and early pregnancy maintenance, they may be particularly vulnerable to environmental endocrine disruptors such as PFASs (4, 5). Consequently, investigating the effects of PFAS on assisted reproduction and their underlying mechanisms is of significant scientific and societal importance (6).

Multiple studies have confirmed an association between PFAS exposure and adverse birth outcomes. For instance, researchers analyzed birth outcomes in the Minneapolis-St. Paul’s area found that individuals with high exposure had significantly lower average birth weights and gestational ages than the control group. These adverse outcomes improved following the installation of water filtration systems (1). Additionally, PFAS may disrupt ovarian function and the fertilization process by affecting the endocrine system and interfering with the balance of reproductive hormones (7, 8). Research indicates that PFAS exposure may be associated with alterations in reproductive endocrine function, with some studies suggesting potential links to conditions such as PCOS (8, 9). Additionally, mixed PFAS exposure may adversely affect embryo quality and impact clinical pregnancy outcomes (10).

Despite these advances, several important knowledge gaps remain in the context of ART populations. First, the number of epidemiological studies specifically conducted in ART cohorts remains limited, and existing studies are often constrained by relatively small sample sizes and heterogeneous study designs. Second, although PFAS are believed to exert their effects by disrupting the follicular microenvironment, direct evidence from human follicular fluid or granulosa cell–based analyzes remains insufficient, limiting mechanistic interpretation (11). Third, most current studies focus on single-compound exposure, whereas real-world exposure occurs as complex mixtures of PFAS and other persistent pollutants, and mixture-based analyzes remain relatively scarce. Finally, findings across studies are not entirely consistent, particularly for clinical endpoints such as implantation and pregnancy outcomes, which may reflect differences in exposure assessment, study populations, and the multifactorial nature of ART success (12).

Therefore, the objective of this review is to systematically synthesize current evidence on the associations between PFAS exposure and ART outcomes, with a particular focus on their effects across different stages of the ART process. This review further aims to integrate epidemiological and mechanistic findings, highlight inconsistencies and methodological limitations in existing studies, and provide a comprehensive framework for understanding how PFAS-induced disruption of the follicular microenvironment may ultimately influence reproductive outcomes in ART settings.

## 2 Characteristics and environmental distribution of perfluorinated compounds

### 2.1 Chemical structure and classification of PFAS

PFAS are a large and structurally diverse class of synthetic fluorinated compounds. Recent PFAS inventories indicate that the number of recognized PFAS structures has expanded substantially, with the U.S. EPA PFASSTRUCT database listing over 21,000 unique structures as of January 2026. This broad definition reflects the substantial heterogeneity within this chemical class and underscores the importance of distinguishing among different PFAS subclasses in toxicological and epidemiological research (13, 14). PFASs are a class of synthetic compounds characterized by a distinct chemical structure, primarily composed of carbon (C) and fluorine (F) atoms. The fundamental chemical skeleton of PFAS can be summarized by the general formula C_n_F_2n+1_–R, where “C_n_F_2n+1_” represents the perfluoroalkyl tail chain, and “R” denotes the connecting functional group or chain segment (such as –COO^-^, –SO_3_^-^, alkoxy, or longer organic linkers/polymeric units). This distinction determines the polarity and ionization behavior of PFAS (14). Based on their terminal functional groups, PFAS are most commonly classified into two major subclasses: perfluoroalkyl carboxylic acids (PFCAs) and perfluoroalkyl sulfonic acids (PFSAs). PFCAs, such as perfluorooctanoic acid (PFOA), contain a carboxylate (–COO^-^) functional group and are widely studied due to their persistence and bioaccumulation potential. In contrast, PFSAs, including perfluorooctane sulfonate (PFOS), contain a sulfonate (–SO_3_^-^) group, which confers even greater environmental persistence and protein-binding affinity (14). These two subclasses represent the most extensively investigated PFAS in human biomonitoring and reproductive epidemiology studies (15). Based on the length of the perfluoroalkyl chain, PFAS are commonly categorized into short-chain and long-chain compounds. Importantly, the definition of long-chain PFAS depends on the functional group: for PFCAs, long-chain compounds are typically defined as those with ≥C7 carbons, whereas for PFSAs, the threshold is ≥C6. Accordingly, commonly studied C8 compounds such as PFOA and PFOS are classified as long-chain PFAS (16). Specific thresholds vary across different studies. Long-chain PFAS (e.g., C8 compounds such as PFOA and PFOS) are generally considered more bioaccumulative and have longer biological half-lives in humans, whereas short-chain PFAS (e.g., PFHxA, PFBS) are more water-soluble and less bioaccumulative but exhibit greater environmental mobility, raising concerns about continuous exposure. The increasing regulatory restrictions on long-chain PFAS have led to a global shift toward the production and use of short-chain alternatives (17). Thus, based on chain length and functional groups, PFAS can be classified into multiple types, including PFCAs and PFSAs. Perfluoroalkyl acids (PFAAs) represent a broader category that includes both PFCAs and PFSAs, as well as related compounds such as perfluoroalkane sulfonamides (FASAs) and fluorotelomer-based substances. These compounds may act as precursors that degrade into terminal PFAAs in environmental and biological systems, thereby contributing to internal exposure burdens (18). The chemical structure of PFAS typically features long fluorinated alkyl chains with strong C-F bonds, conferring exceptional thermal and chemical stability (19). As a result, PFASs are ubiquitous in environmental matrices, and their considerable structural diversity contributes to distinct metabolic fates and toxicological profiles in biological systems (20).

In recent years, a growing class of emerging PFAS alternatives has been introduced to replace legacy compounds such as PFOA and PFOS. These include substances such as hexafluoropropylene oxide dimer acid (HFPO-DA, commonly known as GenX), ammonium 4,8-dioxa-3H-perfluorononanoate (ADONA), and chlorinated polyfluoroalkyl ether sulfonates (e.g., F53-B). Although these compounds have been introduced as alternatives with potentially lower bioaccumulation, emerging experimental and epidemiological evidence suggests that they may still exhibit endocrine-disrupting properties, placental transfer potential, and possible reproductive or developmental toxicity. However, compared with legacy PFAS, their effects on reproductive health and ART-related outcomes remain insufficiently characterized, highlighting an important gap in current research (21, 22). Importantly, this structural and functional diversity has direct implications for ART-related research, as different PFAS subclasses (e.g., PFCAs vs. PFSAs, long-chain vs. short-chain, and emerging alternatives) may exhibit distinct distribution patterns in biological matrices such as serum and follicular fluid, as well as differential associations with ovarian response, embryo quality, and clinical outcomes (23). Therefore, a clear classification framework is essential for interpreting and comparing findings across epidemiological studies.

Notably, most current epidemiological and experimental studies in reproductive toxicology have predominantly focused on legacy PFAS such as PFOA and PFOS, while substantially fewer data are available for other subclasses, including fluorotelomer-based compounds, FASAs, and ether-based alternatives. This imbalance may limit the generalizability of existing findings across the broader PFAS chemical space (16). Furthermore, differences in physicochemical properties, bioaccumulation potential, and biological half-lives across PFAS subclasses may result in distinct toxicokinetic behaviors and tissue distribution patterns. As a consequence, extrapolating reproductive or ART-related effects from well-studied compounds (e.g., PFOA/PFOS) to other PFAS should be approached with caution, highlighting the need for compound-specific and class-specific investigations in future research (24, 25).

### 2.2 Environmental persistence and bioaccumulation of PFAS

PFAS are widely referred to as “forever chemicals” due to their exceptional resistance to environmental degradation. This persistence is primarily attributed to the strong carbon–fluorine (C–F) bonds within their molecular structure, which confer high thermal, chemical, and biological stability. As a result, PFAS are resistant to photolysis, hydrolysis, and microbial degradation, allowing them to persist for extended periods in water, soil, and biota (26). The environmental behavior of PFAS is strongly influenced by their chain length and functional groups. Long-chain PFAS (e.g., PFOS and PFOA) tend to exhibit higher bioaccumulation potential and stronger sorption to organic matter, leading to persistence within food webs and biomagnification across trophic levels. In contrast, short-chain PFAS are generally more water-soluble and mobile, facilitating their widespread distribution in aquatic environments, although they are often considered less bioaccumulative (19). Regarding acid-base properties, most PFCAs and PFSAs possess extremely low pKa values (typically <1) and therefore exist predominantly in ionic form under environmental pH conditions. This ionic nature contributes to their high mobility in aqueous systems and limits their removal through conventional water treatment processes. Consequently, PFAS contamination has become ubiquitous, leading to continuous low-level human exposure through drinking water, diet, and environmental contact (27). These characteristics collectively contribute to the widespread environmental distribution and long-term persistence of PFAS, which in turn support for their bioaccumulation in human populations and raise concerns about chronic exposure and associated health risks.

## 3 Environmental sources, exposure pathways, and ART-relevant exposure to PFAS

### 3.1 Major environmental sources of PFAS

**Figure 1:**
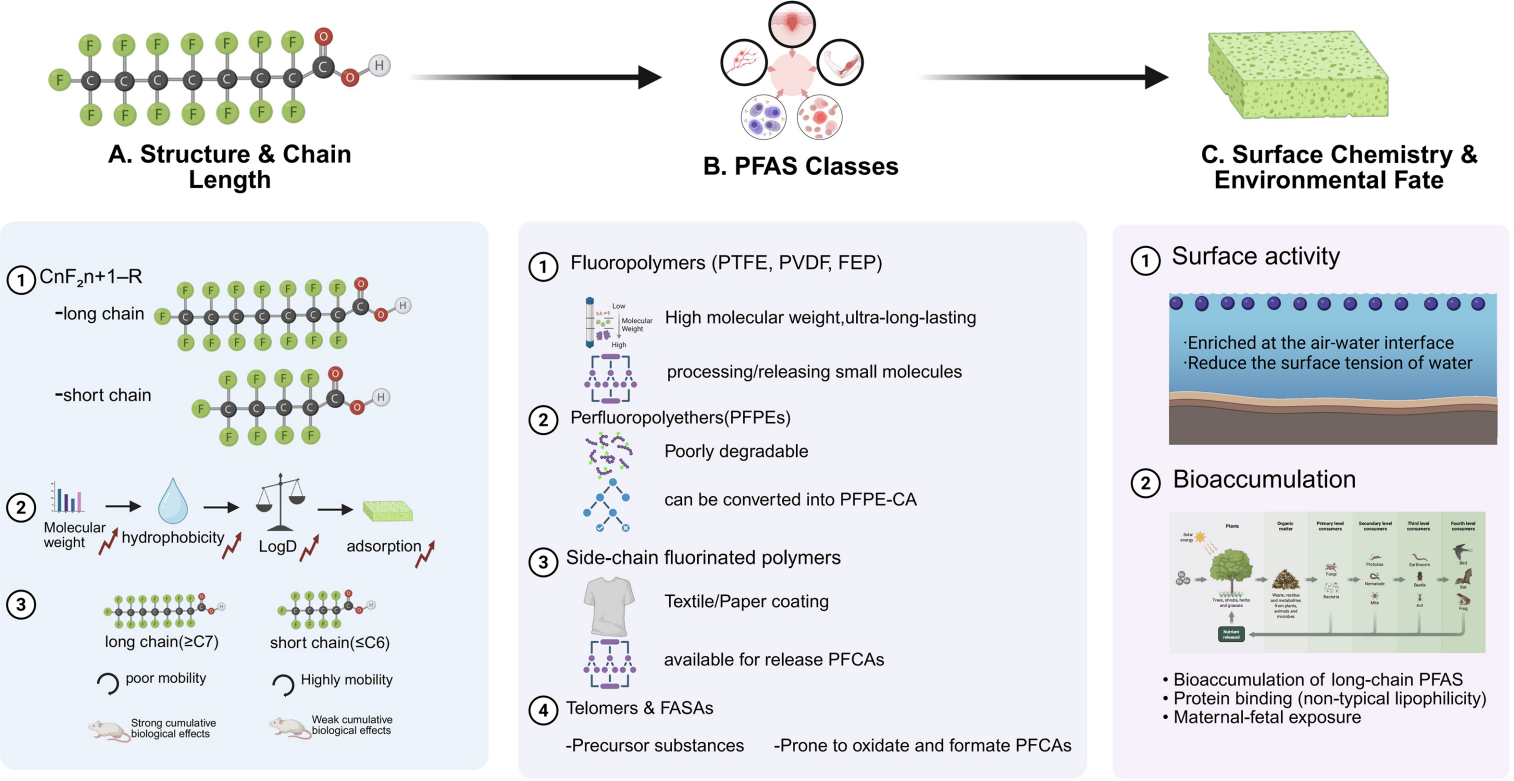
The structure of PFAS determines their physicochemical behavior. **(A)** Structure & Chain Length. PFAS share a characteristic C_n_F_2n+1_–R backbone, where carbon-chain length (e.g., long-chain ≥C7 vs. short-chain ≤C6 species) and terminal functional groups (carboxylates, sulfonates, etc.) govern key physicochemical parameters. Increasing chain length results in higher molecular weight, hydrophobicity, and LogD, leading to greater sorption to solids and reduced mobility. In contrast, short-chain PFAS exhibit higher aqueous mobility but lower bioaccumulative potential. Long-chain PFAS demonstrate stronger protein binding and higher biological persistence, contributing to cumulative health risks. **(B)** PFAS Classes. PFAS include diverse subclasses with distinct structures and environmental behaviors: 1) Fluoropolymers (PTFE, PVDF, FEP) are high–molecular weight, ultra-stable materials that may release small PFAS molecules during production or degradation. 2) PFPEs are poorly degradable and can transform into PFPE-carboxylates. 3) Side-chain fluorinated polymers used in textile and paper coatings can release PFCAs. 4) Telomers and FASAs act as PFAS precursors prone to oxidation into PFCAs. **(C)** Surface Chemistry & Environmental Fate. PFAS exhibit strong surface activity, preferentially enriching at the air–water interface and markedly reducing water surface tension. This behavior promotes their accumulation at environmental interfaces such as soil capillary zones and particle surfaces. Long-chain PFAS readily bind to proteins and biomolecules, facilitating trophic transfer and maternal–fetal exposure. Their combined interfacial behavior and chemical stability contribute to pronounced environmental persistence and bioaccumulation. This figure was created by Biorender.com.

Polymeric and additive PFAS include substances such as fluoropolymers, Perfluoropolyethers (PFPEs), fluorinated side-chain polymers, telomer compounds, and FASAs (**Figure 1**). They constitute a significant subcategory of PFAS, characterized by structural diversity, extensive applications, and complex environmental behavior (28). Fluoropolymers (e.g., PTFE, PVDF) exhibit extreme persistence due to their extremely high molecular weight and fully fluorinated carbon-fluorine backbone, releasing small-molecule PFAS during production and degradation. PFPEs consist of –(CF_2_–O)– repeating units, demonstrating extremely low environmental degradability. Some PFPEs may convert to PFPE carboxylates, but the potential toxicity of these carboxylates remains unclear. Fluorinated side-chain polymers and telomer series substances can release multiple perfluorinated carboxylic acids through environmental oxidation, making them significant “PFAS precursors (29).” FASAs represent a key historical source of PFOS, exhibiting high stability and bioaccumulation potential. Importantly, these precursor compounds serve as continuous secondary sources of terminal PFAS (e.g., PFOA and PFOS) in the environment, contributing to long-term and diffuse contamination beyond direct emissions. These substances are widely used in industrial applications, including lubricants, sealants, and oil- and water-repellent coatings for textiles, paper products, and packaging materials (30). Their environmental inputs primarily stem from industrial production, firefighting foam usage, wastewater treatment plant discharges, and PFAS-containing products employed in agriculture (31). This widespread use and multi-source release result in ubiquitous environmental contamination, forming the primary basis for human exposure across different populations.

### 3.2 Environmental monitoring and distribution of PFAS

In recent years, as concerns over PFAS environmental pollution have intensified, multiple countries and regions have begun implementing monitoring programs to assess their concentrations in water bodies and soil (32). Despite this, current monitoring efforts still face several challenges, including a lack of standardized testing methods and inconsistent data. Many regions have yet to establish effective PFAS monitoring systems, resulting in an insufficient understanding of the true distribution of PFAS in the environment (33, 34). This limitation is further complicated by the presence of polymeric and precursor PFAS, which, despite not always being directly measurable, can undergo environmental transformation into smaller, terminal PFAS compounds. Collectively, these substances combine exceptional persistence with the potential to contribute substantial amounts of small-molecule PFAS, thereby complicating accurate exposure assessment. Consequently, they have become a core focus of group-based regulatory strategies proposed by organizations such as the Organisation for Economic Co-operation and Development (OECD) and the United States Environmental Protection Agency (EPA) (35). Moreover, most conventional monitoring strategies primarily target a limited number of legacy PFAS (e.g., PFOA and PFOS), potentially underestimating total PFAS exposure due to numerous precursors and newly emerging compounds. Recent advances in high-resolution mass spectrometry (HRMS), particularly non-target and suspect screening approaches, have significantly expanded the ability to detect a broader spectrum of PFAS, including previously unrecognized and emerging compounds. Environmental monitoring studies using HRMS have demonstrated that conventional targeted analyzes capture only a fraction of the total PFAS burden, while a substantial proportion of unidentified organofluorine compounds remains undetected in water, food, and environmental samples (36–38). These findings highlight the limitations of traditional targeted methods and underscore the importance of incorporating HRMS-based approaches for more comprehensive exposure assessment. As a result, the true environmental burden of PFAS, particularly in regions without comprehensive monitoring systems, remains likely underestimated.

### 3.3 General population exposure pathways

Exposure to PFAS in the living environment primarily occurs through air, soil, and water sources (**Table 1**). Due to their chemical stability and persistence, PFAS are widely present in a range of products, including cleaning agents, coatings, and food packaging materials. These compounds can be airborne, particularly in industrial areas and locations where PFAS-containing products are used, where airborne PFAS concentrations may significantly increase (39). Cosmetics and personal care products are a significant source of PFAS exposure through the skin (40). Additionally, soil and water contamination can lead to PFAS entering the human body through drinking water and the food chain (41). Epidemiological studies have shown that women residing in PFAS-contaminated areas exhibit consistently elevated PFAS concentrations in their bodies, which may adversely affect reproductive health (42).

**Table 1:** Primary sources and exposure routes of PFAS.

Categories of PFAS	Main sources	Mode of transmission	Reference
Short-chain PFAS ·(≤C6, e.g., PFHxA, PFBS) ·Typical matrices detected: Drinking water, serum (low levels), indoor environment	Landfill leachates	Water pathways (surface runoff, wastewater treatment plant discharges) Transported over long distances via atmospheric wet and dry deposition	[33], [40-43]
	Wastewater treatment plants (WWTPs)		
	Leaky septic tanks		
	Textile, paper and personal care products		
	Human waste from homeless encampments		
	Cosmetics and personal care product (sunscreens, face cream, eye cream)		
Long-chain PFAS ·(≥C7, e.g., PFOA, PFOS, PFNA) • Typical matrices detected: Serum, follicular fluid, placenta	Aqueous Film-Forming Foam (AFFF)	Particulate matter adsorption(soil) Bioconcentration along the food chain Maternal transmission	[33], [40-43]
	Metal plating		
	PFAS manufacturing facilities		
	Textile and paper coating operations		
	Face cream and hair care products		

Food and drinking water represent major exposure pathways for PFASs in the general population. Studies indicate that certain seafood and animal products—such as fish, shellfish, and meat—contain elevated PFAS concentrations, particularly in aquatic species raised in contaminated waters. Drinking water contamination remains a critical concern, especially in areas where PFAS-containing firefighting foams have been used. PFAS levels in drinking water are closely linked to health risks for residents, particularly reproductive health risks for women (43). Additionally, the methods used to treat and filter drinking water can affect the effectiveness of PFAS removal. Unfiltered water sources may contain higher concentrations of PFAS, increasing the population’s exposure risk (44). Epidemiological evidence indicates that individuals residing in contaminated regions exhibit consistently higher internal PFAS levels, highlighting the significance of chronic low-dose exposure in shaping body burden.

### 3.4 ART-specific exposure pathways and clinical relevance

In addition to general environmental exposure, individuals undergoing ART may experience additional, procedure-related PFAS exposure. Medical devices and laboratory materials used in ART procedures may contain PFAS-based coatings designed to provide hydrophobic and anti-fouling properties. These materials include plasticware, tubing, culture dishes, and other consumables routinely used during oocyte retrieval, fertilization, and embryo culture. PFAS may be released from these materials into biological samples during clinical procedures, potentially contributing to localized exposure within the reproductive microenvironment (45). Given that ART involves highly controlled and sensitive *in vitro* conditions, even low-level contamination from laboratory materials may disproportionately affect oocyte quality, embryo development, and implantation potential. Furthermore, PFAS exposure may disrupt endocrine signaling and hormonal balance, thereby influencing ovarian response and pregnancy outcomes (46). Therefore, ART represents a unique exposure scenario in which both environmental and procedure-related sources may converge, underscoring the need for stricter quality control of medical materials and improved awareness of potential contamination pathways. These exposure pathways provide the foundation for internal PFAS accumulation in human tissues, particularly within reproductive compartments, which will be discussed in the following section.

## 4 Human internal exposure, reproductive distribution, and implications for ART outcomes

### 4.1 Internal exposure and systemic distribution of PFAS

PFAS exhibit typical surface-active characteristics, significantly reducing water’s surface tension— a property especially pronounced in PFAS with longer chains and sulfonic acid terminal groups. This surface tension-reducing ability not only leads to high PFAS accumulation at air–water interfaces and soil capillary interfaces but also enhances their adsorption tendency on biofilms, cell membranes, and protein surfaces. Importantly, PFAS display strong binding affinity for serum proteins such as albumin, which facilitates their systemic transport and contributes to their prolonged biological half-lives in humans (47). This protein-binding behavior represents a key determinant of PFAS toxicokinetics, distinguishing them from other persistent organic pollutants that preferentially accumulate in adipose tissue. As a consequence, PFAS can distribute extensively within the human body, accumulating in blood, liver, and, critically, reproductive tissues and fluids, including follicular fluid and seminal plasma (48, 49). In addition to female reproductive compartments, increasing evidence indicates that PFAS are also detectable in male reproductive matrices, including seminal plasma and spermatozoa. Human biomonitoring studies have identified both legacy and emerging PFAS in semen, with correlations observed between serum and seminal concentrations, suggesting systemic transfer and accumulation within the male reproductive system (50, 51). Advanced analytical approaches, including LC-HRMS–based screening, have further revealed a broader spectrum of PFAS and related contaminants in human semen, highlighting the complexity of male reproductive exposure (52). Epidemiological studies have reported associations between PFAS exposure and multiple sperm quality parameters, including reduced sperm concentration, decreased motility, and altered morphology (53, 54). Sperm motility appears to be one of the more sensitive indicators of PFAS exposure, with several studies reporting negative associations between PFAS concentrations and progressive motility, which may impair the ability of sperm to reach and penetrate the oocyte (53, 55). Associations with sperm concentration and morphology have also been reported, although findings remain inconsistent across studies (56, 57). These observations suggest that the male reproductive system represents an additional target of PFAS exposure, with potential implications for fertilization processes relevant to ART. Notably, the detection of PFAS in follicular fluid provides direct evidence that developing oocytes are exposed to these compounds within the ovarian microenvironment, thereby establishing a mechanistic link between environmental exposure and potential reproductive toxicity. These physicochemical characteristics suggest that PFAS can directly interact with hormonally active microenvironments that are critical for ART, including ovarian follicles, developing oocytes, and early embryos (58) Consequently, chronic low-level PFAS exposure is widespread, including among women undergoing ART, Given the tightly regulated hormonal and metabolic conditions required during ovarian stimulation, oocyte retrieval, fertilization, and embryo transfer, even subtle disruptions caused by persistent environmental contaminants may have disproportionate effects on ART outcomes. This raises concerns regarding continuous internal exposure during critical procedural windows (59). In summary, the structural stability, surface activity, and ionization state of PFAS collectively determine their resistance to environmental degradation and their tendency to accumulate within living organisms, posing potential risks to ecosystems and human health (60). Existing research has confirmed that PFAS accumulate in soil, water bodies, food chains, and mammalian organisms. Among these, long-chain PFAS (such as PFOS and PFOA) typically exhibit greater bioaccumulation potential and demonstrate pronounced biomagnification effects within food chains (61, 62). Such biomagnification may further amplify internal exposure levels in humans, particularly during sensitive reproductive windows, thereby increasing vulnerability to reproductive dysfunction and adverse ART outcomes (63).

Taken together, these physicochemical and toxicokinetic characteristics not only govern the systemic distribution and persistence of PFAS within the human body but also contribute to substantial inter-individual variability in internal exposure levels. At the population level, such variability is further influenced by environmental and occupational factors. Biomonitoring studies have shown that individuals residing in areas with known PFAS contamination sources—such as regions affected by aqueous film-forming foam (AFFF) use, including military bases and airports—often exhibit markedly elevated serum concentrations of legacy PFAS, particularly PFOA and PFOS, compared with the general population (64, 65). These findings suggest that external exposure scenarios can significantly amplify internal PFAS burdens, potentially leading to higher concentrations in biologically relevant compartments, including reproductive tissues and fluids. However, while such high-exposure populations provide important insight into PFAS toxicokinetics and systemic accumulation, direct evidence linking these exposure gradients to ART-specific outcomes remains limited, highlighting an important gap for future investigation.

### 4.2 Accumulation in reproductive compartments and microenvironmental relevance

**Figure 2:**
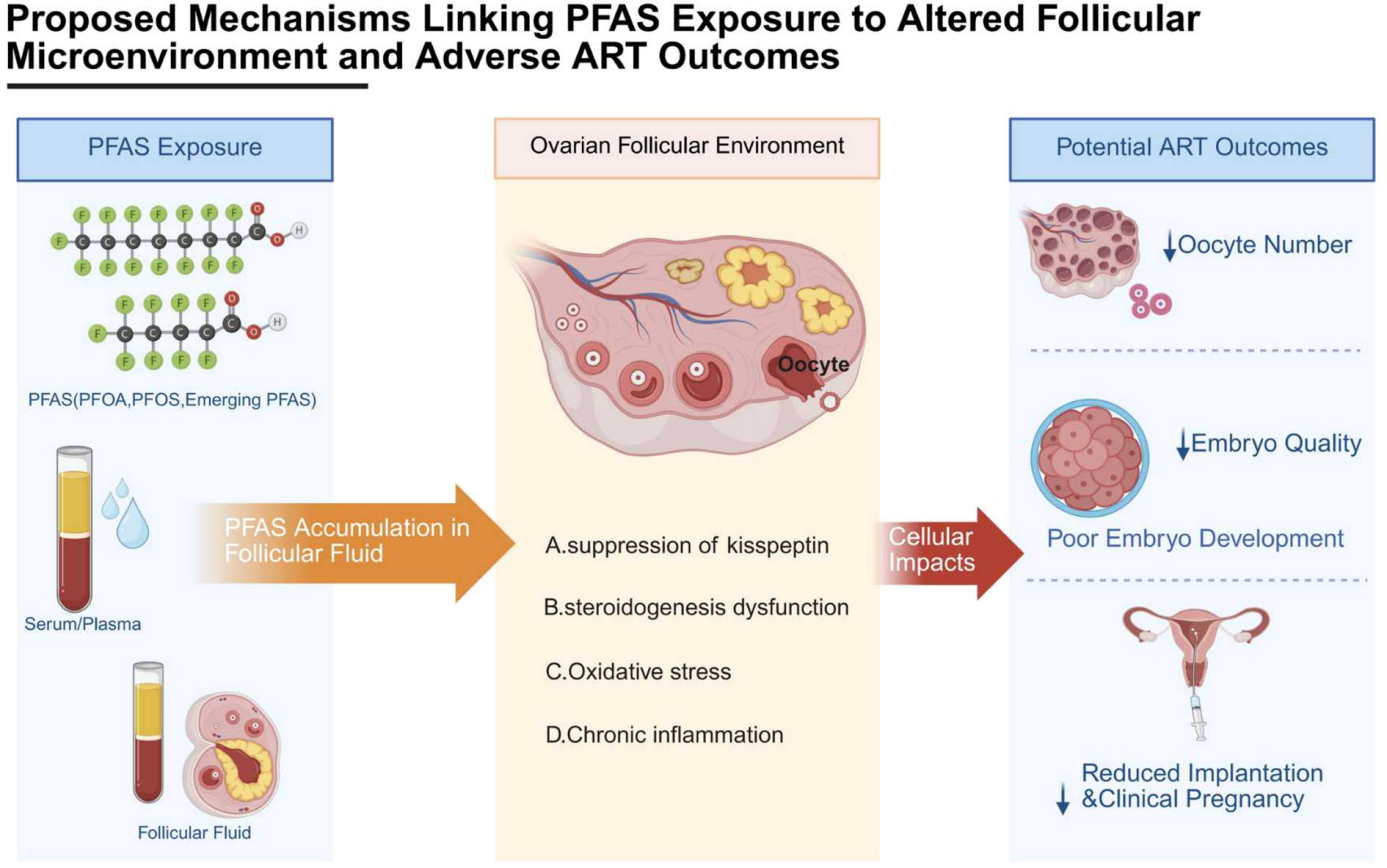
Proposed mechanisms linking PFAS exposure to adverse outcomes in assisted reproductive technologies (ART). PFAS, including commonly detected compounds such as PFOA and PFOS, can accumulate in human biological matrices such as serum, plasma, and follicular fluid. These persistent chemicals may reach the ovarian follicular microenvironment through systemic circulation and cross the blood–follicle barrier, thereby directly exposing developing oocytes. Within the follicular environment, PFAS exposure has been associated with several cellular and metabolic disturbances, including suppression of kisspeptin, steroidogenesis dysfunction and oxidative stress, and chronic inflammation. These molecular disruptions may impair oocyte competence and early embryo development, potentially leading to reduced oocyte yield, decreased embryo quality, and lower implantation or clinical pregnancy rates in women undergoing assisted reproductive technologies. The diagram summarizes current epidemiological and experimental evidence on how PFAS exposure may influence reproductive outcomes during ART treatment. This figure was created by Biorender.com.

PFAS, persistent environmental pollutants, may impair ART outcomes by disrupting the follicular microenvironment (**Figure 2**). Researchers have shown significant interest in the association between PFAS exposure and ART outcomes. Epidemiological evidence consistently indicates that PFAS exposure is associated with alterations in early ART endpoints, including reduced numbers of retrieved oocytes, mature oocytes, and high-quality embryos, whereas associations with implantation rate, clinical pregnancy, and miscarriage remain inconsistent (66). These findings suggest that PFAS primarily affects early stages of the reproductive process, such as oocyte competence, embryo quality, and follicular microenvironmental regulation, rather than exerting uniform effects on downstream clinical outcomes (4). Consistently, PFAS has been implicated in impaired oocyte maturation, embryo development, and endocrine function (67). In parallel, evidence from epidemiological studies indicates that prenatal PFAS exposure disrupts maternal–fetal metabolic homeostasis and placental function, and is associated with an increased risk of pregnancy complications (68, 69). However, as these findings are largely derived from general population studies, their direct relevance to ART populations should be interpreted with caution. Notably, current evidence does not consistently support an association between PFAS exposure and reduced pregnancy rates or increased miscarriage risk in ART settings. One of the most consistently reported associations is between PFAS exposure and gestational hypertension (GH) and preeclampsia (PE) (70). Multiple prospective cohort studies suggest that early pregnancy exposure to PFAS is associated with an increased risk of GH/PE, although causality remains to be fully established (71). The underlying biological mechanisms are not yet fully elucidated, but may involve impaired endothelial function, oxidative stress, inflammatory activation, and altered placental vascular remodeling (72). These pathways are supported by experimental and mechanistic studies, but direct *in vivo* evidence linking PFAS exposure to a complete preeclampsia-like phenotype remains limited. PFAS have been shown to impair placental angiogenesis in experimental models, potentially through the induction of oxidative stress and disruption of VEGF signaling pathways, which may contribute to a pro-hypertensive milieu and adverse pregnancy outcomes (73). Due to the aforementioned maternal effects and placental toxicity, multiple cohort studies have observed increased risks of fetal growth restriction (FGR), low birth weight (LBW), and preterm birth (74). Additionally, maternal nutritional status (such as folate and BMI) was observed to modulate the effects of PFAS on fetal weight, suggesting the potential existence of susceptible populations (75). In a prospective cohort study of 336 women who conceived through ART, multiple PFAS were associated with an increased risk of gestational diabetes, which in turn affected pregnancy outcomes (76, 77). Available evidence from experimental studies indicates that PFAS exposure can activate PPARα-dependent pathways and alter lipid metabolism, leading to hepatic lipid accumulation. In addition, PFAS has been shown to disrupt glucose homeostasis and insulin signaling in animal models. However, direct evidence linking PFAS exposure to pancreatic β-cell dysfunction or pregnancy-specific insulin resistance remains limited (78–80). This may also be related to PFAS disrupting the regulation of maternal thyroid hormones and thyroid-stimulating hormone (TSH), thereby affecting glucose homeostasis and increasing the risk of GDM (81). Epidemiological studies have reported associations between PFAS exposure and an increased risk of miscarriage, although findings remain inconsistent across populations (82). Potential mechanisms may involve endocrine disruption and altered immune regulation, but direct mechanistic evidence linking these pathways to pregnancy loss remains limited (83). During the embryo implantation phase, PFAS may influence embryo–uterine interactions by modulating the local immune microenvironment. Experimental studies have shown that PFAS exposure can alter the expression of inflammatory mediators, such as IL-6 and TNF-α, in reproductive and placental cell models. Given the critical role of immune tolerance in implantation, these alterations may potentially interfere with embryo–endometrium crosstalk. However, direct evidence linking PFAS-induced immune dysregulation to implantation failure or early pregnancy loss remains limited (84, 85). Taken together, these findings suggest that PFAS may interfere with the finely tuned embryo–endometrium crosstalk; however, whether such molecular and cellular alterations translate into measurable impairments in implantation or clinical pregnancy outcomes in ART settings remains to be fully established.

### 4.3 Implications for ART outcomes and offspring health

Exposure to PFAS has been associated with a range of adverse perinatal outcomes, although the strength and consistency of evidence vary across endpoints. Epidemiological studies have most consistently reported associations between prenatal PFAS exposure and reduced birth weight, as well as an increased risk of preterm birth (86, 87). In contrast, evidence linking PFAS exposure to congenital anomalies remains limited and inconsistent, with some studies suggesting potential associations but lacking reproducibility across populations. Similarly, while several cohort studies have explored potential effects of PFAS on neurodevelopment, including cognitive function and behavioral outcomes, findings are heterogeneous and do not yet support definitive conclusions (88, 89). Overall, current evidence suggests that PFAS exposure during pregnancy may have implications for fetal growth and child development; however, the magnitude, specificity, and long-term clinical significance of these effects remain to be fully established (90, 91). These uncertainties highlight the need for cautious interpretation and further well-designed longitudinal studies, particularly in ART populations.

## 5 Evidence linking PFAS exposure to ART outcomes

Over the past decade, a growing number of epidemiological studies have investigated the potential effects of PFAS on reproductive outcomes in women undergoing ART. These studies, summarized in **Table 2**, have primarily been conducted in IVF or IVF/ICSI cohorts in China, Europe, the United States, and Australia. Most investigations use prospective cohort designs and measure PFAS concentrations in biological matrices such as serum, plasma, or follicular fluid, allowing the evaluation of internal exposure levels during ART treatment. Importantly, PFAS represent a large class of chemicals rather than a single compound. As shown in **Table 3**, most ART studies have measured several legacy PFAS, particularly PFOA and PFOS, which belong to the two major PFAS subclasses: PFCAs and PFSAs. Some recent studies have also begun to examine emerging PFAS replacements, reflecting the evolving exposure landscape. Together, these studies provide important insights into how PFAS exposure may influence ovarian function, embryo development, and pregnancy outcomes in ART populations. However, some discrepancies remain in the findings across studies, which may be attributed to differences in sample size, PFAS types, exposure assessment methods, and statistical models.

**Table 2:** Summary of epidemiological studies on PFAS exposure and ART outcomes.

Study	Year	Country	Study design	Sample size	PFAS matrix	Statistical model	Key findings	Special features
Petro et al.	2014	Belgium	Prospective IVF cohort	~38 women	Blood + follicular fluid	Single: Logistic regression	Higher PFAA-contamination is associated with a better fertilization rate in ART.	First evidence of PFAS contamination in human follicular fluid
McCoy et al.	2017	USA	Observational cohort	~50 women	Blood + follicular fluid	Single: Pearson correlation analyses	PFAS associated with ovarian function markers but no significant association with implantation or blastocyst formation	Simultaneous measurement in blood and follicular fluid
Kim et al.	2020	Australia	Cross-sectional infertility cohort	~97 women	Follicular fluid	Single: Linear regression model	Multiple PFAS detected in follicular fluid and there was no relationship between PFAS and ferlitisation rate	Early evidence from Australian IVF population
Ma et al.	2021	China	Prospective IVF cohort	~96 couples	Parental plasma	Single: Logistic regression/Poisson regression models	Parental PFAS exposure was associated with oocyte yield, fertilization, and embryo quality, but not with implantation, clinical pregnancy, or live birth after IVF.	Evaluated both maternal and paternal PFAS exposure
Hong et al.	2022	China	Prospective IVF-ET cohort	~124 women	Blood + follicular fluid	Single: Logistic regression/Poisson regression models	Demonstrated PFAS transfer from blood to follicular fluid; most PFAS not significantly associated with pregnancy outcomes	Blood–follicular transfer analysis
Lefebvre et al.	2022	France	Prospective IVF cohort	~136 women	Blood + follicular fluid	Single: Poisson regression model Mixture: BKMR model	No statistically significant associations between PFAS levels and intermediary or clinical IVF outcomes.	Multi-pollutant epidemiological framework
Bj¨orvang et al.	2022	Sweden	Observational ART cohort	~185 women	Blood + follicular fluid	Single: Logistic regression/Linear regression models Mixture: WQS regression	Persistent organic pollutants including PFAS associated with altered reproductive outcomes	Studied multiple classes of environmental pollutants
Zeng et al.	2023	China	Prospective IVF cohort	~729 women	Follicular fluid	Single: RCS model Mixture: BKMR model	Higher PFAS concentrations associated with lower embryo quality	729 follicular fluids is the largest reported to date,far eclipsing the sample sizes of previous studies
Bellavia et al.	2023	Sweden & Estonia	Multicenter ART cohort	~333 women	Follicular fluid	Single: Linear regression/Logistic regression models Mixture: BKMR regression	Chemical mixtures may interfere with ovarian sensitivity in women.	One of the first studies applying mixture modeling in ART exposure research
Shen et al.	2024	China	Prospective IVF cohort	~259 women	Blood	Single: Logistic regression/GLM models Mixture: BKMR model	PFAS exposure was associated with reduced fertilization and fewer high-quality embryos, but not with implantation or pregnancy outcomes after IVF.	Detailed analysis of fertilization and embryo quality endpoints
Xu et al.	2025	China	Prospective IVF cohort	~378 women	Follicular fluid	Single: Beta regression/Logistic regression models Mixture : BKMR model/Quantile-based g-computation model	Higher PFOA associated with poorer embryo quality and altered lipid and energy metabolism pathways	Integration of metabolomics with environmental exposure analysis
Boney et al.	2026	USA	Prospective IVF cohort	~86 women	Follicular fluid	Single: Descriptive + Shapiro-Wilk test + t-tests	Both legacy and emerging PFAS detected in follicular fluid	First detection of emerging PFAS replacement compounds in IVF follicular fluid in NC serum
Yang et al.	2026	China	Prospective IVF/ICSI cohort	~275 women	Blood	Single: Logistic regression/Poisson regression models Mixture: QGC model	PFAS exposure was associated with reduced oocyte and embryo metrics, but not with fertilization or pregnancy outcomes.	Using the QGC model to assess the impact of PFAS mixtures on embryo quality and identified the PFAS that contributed most to the exposure–response relationship

**Table 3:** Biomonitoring studies of PFAS in ART populations.

Study	Biological matrix	Detected PFCA (major compounds)	Detected PFSA (major compounds)	Detected emerging/other PFAS	Representative concentration	Number of PFAS analyzed
Petro 2014	Blood + follicular fluid	PFOA, PFNA	PFOS, PFHxS	–	PFOS: 2.8–12.5ng/mL(Blood), 0.1–30.4ng/mL(FF); PFOA: 1.0–3.2ng/mL(Blood), 0.3–3.3ng/mL(FF)	~14
McCoy 2017	Blood + follicular fluid	PFOA, PFNA, PFDA,PFHpA	PFOS, PFHxS	–	PFOS: 6.52 ± 0.50 ng/g (Blood), 5.33 ± 0.42 ng/g(FF); PFOA: 2.44 ± 0.30 ng/g (Blood), 1.94 ± 0.20ng/g(FF)	~15
Kim 2020	Follicular fluid	PFOA, PFNA, PFDA,PFHpA	PFOS, PFHxS,PFHpS	–	PFOS: 0.7–22.4ng/mL(FF),PFOA:0.3–14.5ng/mL(FF)	~32
Ma 2021	Parental plasma	PFOA, PFNA, PFDA,PFUA	PFOS, PFHxS	PFOSA	PFOS:2.1-146.2 ng/mL(male plasma), 0.8-64.9 ng/mL (female plasma); PFOA: 6.0-86.4 ng/mL(male plasma), 2.1-71.0 ng/mL (female plasma)	~10
Hong 2022	Blood + follicular fluid	PFOA, PFNA, PFDA,PFHpA,	PFOS, PFHxS,PFHpS	Additional PFAS analogues(6:2 Cl-PFESA)	PFOS: 1.67-15.24 ng/mL(Blood), 0.72-12.71 ng/mL(FF); PFOA: 1.36-82.99 ng/mL(Blood),1.18-83.79 ng/mL(FF)	~45
Lefebvre 2022	Blood (PFAS); Follicular fluid (other pollutants)	PFOA,PFNA, PFDA,PFUnA	PFOS, PFHxS	Other persistent pollutants	PFOS: 1.42–2.52 ng/g(Blood), PFOA:0.80–1.56 ng/g(Blood)	~14
Bj¨orvang 2022	Blood + follicular fluid	PFOA, PFNA, PFDA,PFHpA	PFOS, PFHxS	Other persistent pollutants	Relative proportion reported (no absolute concentration data)	~8
Zeng 2023	Follicular fluid	PFOA, PFNA, PFDA,PFUnDA	PFOS, PFHxS,PFHpS	–	PFOS: 1.04–2.61 ng/mL(FF),PFOA:0.59–1.71 ng/mL(FF)	~32
Bellavia 2023	Follicular fluid	PFOA, PFNA, PFDA,PFUnDA	PFOS, PFHxS	Other persistent pollutants	PFOS: 0.17–15.05 ng/mL(FF),PFOA:0.17–8.05 ng/mL(FF)	~6
Shen 2024	Blood	PFOA, PFNA, PFDA,PFHpA	PFOS, PFHxS	–	PFOS: 0.007–320.752 ng/mL(Blood), PFOA:3.339–62.296 ng/mL(Blood)	~8
Xu 2025	Follicular fluid	PFOA, PFDA, PFNA,PFHpA	PFOS, PFHxS,PFHpS	Additional PFAS analogues(6:2 Cl-PFESA)	PFOS: 1.910-4.840ng/mL(FF),PFOA:5.090-8.590ng/mL(FF)	~29
Boney 2026	Follicular fluid	PFOA, PFNA, PFDA,PFHpA	PFHxS,PFOS, PFHpS	GenX and other replacement PFAS	PFOS: 0.5-12.8 ng/mL(FF),PFHxS:0.5-12.8 ng/mL(FF)	~23
Yang 2026	Blood	PFOA, PFNA, PFDA,PFHpA	PFOS, PFHxS,PFHpS	Additional PFAS analogues(6:2 Cl-PFESA)	PFOS: 1.30–143.72 ng/mL(Blood), PFOA:0.98–26.07 ng/mL(Blood)	~17
Marchiandi2024	Blood + Follicular fluid + Seminal fluid	PFOA, PFBA, PFPeA,PFHpA	PFOS, PFHxS	6:2 FTSA,PFBS	PFOS:0.38-2.91 ng/mL(FF), PFHxS:0.07-1.92 ng/mL (FF); PFBA: 0.02-0.09 ng/mL(SF), 6:2 FTSA:0.07-0.09 ng/mL (SF)	~72

### 5.1 PFAS occurrence and transfer in ART populations

One of the earliest questions addressed by ART studies was whether PFAS can reach the ovarian follicular environment, where oocyte maturation occurs. Early work by Petro et al. (2014) demonstrated that several PFAS, including PFOA and PFOS, could be detected in human follicular fluid, providing the first direct evidence that persistent fluorinated compounds can accumulate in the ovarian microenvironment (92). Subsequent studies confirmed these findings across multiple populations. For example, McCoy et al. (2017) measured PFAS concentrations in both blood and follicular fluid and reported significant correlations between the two matrices, suggesting systemic circulation as a major exposure source (93). Similarly, Hong et al. (2022) further characterized the blood–follicular transfer of PFAS in women undergoing IVF-ET treatment in China. Their results indicated that several PFAS detected in serum could also be detected in follicular fluid, supporting the hypothesis that circulating PFAS can cross the blood–follicle barrier and directly expose developing oocytes (94). In addition to legacy PFAS, recent investigations have begun to identify emerging PFAS replacement compounds in ART populations. For instance, Boney et al. (2026) reported the presence of both legacy PFAS (e.g., PFOA and PFOS) and emerging PFAS, including hexafluoropropylene oxide dimer acid (GenX) and related replacement compounds, in follicular fluid from IVF patients in the United States (95). These findings highlight the importance of considering a broad range of PFAS compounds when evaluating reproductive toxicity.

### 5.2 PFAS exposure and ovarian response

Several epidemiological studies have examined whether PFAS exposure is associated with ovarian response to controlled ovarian stimulation, a key determinant of ART success (93, 96). Indicators of ovarian response typically include the number of retrieved oocytes, mature oocyte rate, and hormonal markers of ovarian reserve. However, findings regarding the association between PFAS exposure and ovarian response remain inconsistent across studies. While several investigations have reported negative associations, including reduced oocyte yield and impaired ovarian function parameters, others have observed no significant relationships between PFAS concentrations and markers of ovarian response. For example, some cohort studies did not detect significant associations between PFAS exposure and the number of retrieved oocytes or related indicators, suggesting potential heterogeneity in observed effects (93). These inconsistencies may reflect differences in study populations, exposure levels, PFAS compound profiles, and methodological approaches. In addition, variability in controlled ovarian stimulation protocols and individual patient characteristics may further contribute to the heterogeneity of findings. Collectively, current evidence suggests that while PFAS may influence ovarian response, the magnitude and direction of this effect are not yet fully consistent across ART cohorts. In addition to conventional indicators such as the number of retrieved oocytes, some studies have incorporated more refined measures of ovarian responsiveness, including the ovarian sensitivity index (OSI), which reflects the efficiency of ovarian response relative to the administered gonadotropin dose. These indices provide a more nuanced assessment of ovarian function during controlled ovarian stimulation (97). Emerging evidence suggests that PFAS exposure may influence not only oocyte yield but also the functional responsiveness of the ovary to exogenous hormonal stimulation. While direct evidence linking PFAS exposure to OSI remains limited, several ART cohort studies have reported associations between PFAS concentrations and reduced oocyte numbers, supporting the hypothesis of impaired ovarian responsiveness (96).

Notably, Ma et al. (2021) extended this perspective by simultaneously assessing PFAS concentrations in both maternal and paternal plasma. Their findings indicated that parental PFAS exposure was associated with altered IVF outcomes, including reduced oocyte yield, highlighting that PFAS-related reproductive toxicity may involve not only ovarian dysfunction but also paternal contributions. This dual-exposure framework represents an important advancement beyond traditional female-centric models and suggests that PFAS may affect ART outcomes through combined effects on both gamete quality and the ovarian response to stimulation (66). Given that ovarian responsiveness determines the pool of available oocytes for subsequent fertilization and embryo development, PFAS-induced alterations at this stage may have downstream consequences for embryo quality and overall ART success.

### 5.3 PFAS exposure and embryo development

Beyond ovarian response, increasing attention has been given to the potential impact of PFAS exposure on embryo development and quality, which are critical predictors of successful implantation and pregnancy. Evidence linking PFAS exposure to embryo development has become increasingly consistent across recent studies. For example, Zeng et al. (2023) and Shen et al. (2024) reported that higher PFAS concentrations were associated with reduced embryo quality and lower fertilization success (45, 58). Importantly, McCoy et al. (2017) provided early evidence of compound-specific effects at the embryo development stage. While most PFAS were not associated with later pregnancy outcomes, higher concentrations of long-chain PFAS, including PFDA and PFUnA, in follicular fluid were inversely associated with blastocyst formation rates (93). These findings suggest that PFAS exposure may exert more pronounced effects during early embryo development than at later stages, such as implantation or clinical pregnancy, and that these effects may depend on specific PFAS compounds (4). Mechanistic insights have also been provided by studies integrating metabolomics approaches. One cohort study examining PFOA found that elevated PFOA levels in follicular fluid were associated with poor embryo quality and alterations in lipid and energy metabolism pathways. These findings suggest that PFAS exposure may disrupt key metabolic processes involved in oocyte maturation and early embryo development (10). Collectively, evidence from multiple studies, including McCoy et al., suggests that PFAS exposure may impair embryo developmental competence in a compound-specific manner, particularly affecting blastocyst formation.

### 5.4 PFAS exposure and ART pregnancy outcomes

Compared with earlier ART endpoints, evidence linking PFAS exposure to implantation rate and clinical pregnancy outcomes remains limited and largely inconclusive. Most studies reported that the majority of PFAS compounds were not significantly associated with implantation rate or clinical pregnancy following IVF-ET treatment (4, 45). However, this apparent lack of association should not be interpreted as definitive evidence of no effect. Across studies, heterogeneity persists, with some reporting compound-specific or subgroup-specific associations, while others observe null findings (67). This pattern suggests that the impact of PFAS on ART success may be subtle and context-dependent rather than uniformly absent. In contrast to upstream endpoints such as oocyte yield and embryo quality, which reflect more direct biological interactions within the follicular microenvironment, implantation and clinical pregnancy outcomes are influenced by a complex interplay of embryonic competence, endometrial receptivity, and clinical factors. As a result, potential PFAS-related effects may be attenuated or masked at these later stages. Notably, several studies that identified associations between PFAS exposure and reduced oocyte or embryo quality did not observe corresponding effects on clinical pregnancy outcomes, further highlighting a possible disconnect between early biological perturbations and ultimate ART success (98). This discrepancy underscores the importance of examining intermediate endpoints when evaluating environmental exposures in ART populations.

### 5.5 PFAS exposure and male reproductive parameters

While most ART-related studies have focused on female reproductive outcomes, emerging evidence suggests that PFAS exposure may also influence male reproductive parameters that are critical for ART success. Epidemiological studies have reported associations between PFAS concentrations in serum or semen and alterations in sperm quality, including sperm concentration, motility, and morphology (56, 57). To provide additional context, we summarized available biomonitoring studies of PFAS in male populations (**Table 4**), which include measurements in serum and/or seminal fluid; however, most of these studies were conducted in general populations rather than ART-specific cohorts. These parameters are key determinants of fertilization efficiency and early embryo development in ART. However, compared with female-focused research, studies on PFAS exposure and male fertility remain limited and inconsistent. Notably, direct evidence linking PFAS levels in semen to ART-specific outcomes (e.g., fertilization rate, embryo quality, or clinical pregnancy) is lacking, leaving the contribution of male exposure insufficiently characterized and representing an important gap for future research.

**Table 4:** Biomonitoring studies of PFAS in human semen and male reproductive outcomes.

Study	Male participants	Study type	Biological matrix	Detected PFAS (major compounds)	Representative concentration	Main findings
Cui et al., 2020.	~651	Biomonitoring + Epidemiological	Serum + semen	PFCA: PFOA, PFNA, PFHpA PFSA: PFOS, PFBS, PFHxS Emerging PFAS: 6:2 Cl-PFESA	PFOS: 1.47-392.01 ng/mL(Serum), <LOQ-9.90 ng/mL(Semen); PFOA: 1.66-95.69 ng/mL(Serum), 0.04-2.97 ng/mL(Semen)	PFAS were detected in both serum and semen; serum–semen distribution patterns differed, and PFAS exposure was associated with male reproductive hormone alterations.
Luo et al., 2022.	~740	Mixture analysis (epidemiological)	Serum	PFCA: PFOA, PFNA, PFHpA PFSA: PFOS, PFBS, PFHxS Emerging PFAS: 6:2 Cl-PFESA	PFOS: 9.1–20.1 ng/mL(Serum) PFOA: 17.9–36.4 ng/mL(Serum)	Mixture-based analysis linked PFAS exposure to semen quality indicators; highlights relevance of both legacy and emerging PFAS in male fertility assessment.
Pan et al., 2023.	~664	Biomonitoring	Serum + semen	PFCA: PFOA, PFNA, PFHpA PFSA: PFOS, PFBS, PFHxS Emerging PFAS: 6:2 Cl-PFESA	PFOS: 1.256–337.0 ng/mL(Serum), <LOQ–8.716 ng/mL(Semen); PFOA: 1.660–95.69 ng/mL(Serum), 0.043–2.966 ng/mL(Semen)	Demonstrated occurrence of both emerging and legacy PFAS in matched serum and semen, supporting transfer into male reproductive fluids and relevance for semen quality.
Petersen et al., 2022.	~1041	Epidemiological (cross-sectional)	Serum	PFCA: PFOA, PFNA, PFHpA PFSA: PFOS, PFBS, PFHxS	PFOS: 2.06–7.76 ng/mL(Serum) PFOA: 0.66–2.18 ng/mL(Serum)	PFAS exposure is not consistently associated with semen quality or testicular volume, but is mildly associated with elevated FSH, suggesting potential reproductive endocrine disruption.
Sánchez-Resino et al., 2023.	~10	Methodological (HRMS-based)	Semen	PFCA: PFOA PFSA: PFOS, PFBS plus other organic contaminants	PFOS: 0.14–1.42 ng/mL(Semen) PFOA: 0.01–0.04 ng/mL(Semen)	Plastic additives and PFAS are commonly found in the semen of men in chemical industrial areas and showed that LC-HRMS can broaden semen biomonitoring beyond conventional targeted panels.
Shi et al., 2025.	~1206	Epidemiological	Semen	PFCA: PFOA, PFNA, PFHpA PFSA: PFOS, PFBS, PFHxS Emerging PFAS: 6:2 Cl-PFESA	PFOA: Median 0.191 ng/mL, with the highest in the case group reaching 0.214 ng/mL (oligoasthenospermia group) PFOS: Median 0.249 ng/mL, with the highest in the case group reaching 0.290 ng/mL (oligoasthenospermia group)	Semen PFAS exposure (especially 6:2 Cl-PFESA and PFHxS) is positively associated with the risk of oligospermia, and antioxidant nutritional supplementation can mitigate this toxic effect.
Sun et al., 2025.	~1019	Epidemiological (longitudinal)	Serum + semen	PFCA: PFOA, PFNA, PFHoA PFSA: PFOS, PFBS, PFHxS Emerging PFAS: 6:2 Cl-PFESA	PFOS: 1.047-7.532 ng/mL(Serum), 0.101-0.411 ng/mL(Semen); PFOA: 1.362-2.556 ng/mL(Serum), 0.076-0.213 ng/mL(Semen)	PFAS in semen were associated with repeated measures of semen quality in healthy adult men, supporting longitudinal relevance of seminal PFAS biomarkers.
Wang et al., 2023.	~2190	Epidemiological	Serum	PFCA: PFOA, PFNA. PFSA: PFOS, PFBS	Not mentioned	PFNA and PFOA exposure may reduce sperm forward motility
Marchiandi 2024	30 couples	Preconception cohort	Blood + Follicular fluid + Seminal fluid	PFCA: PFOA, PFBA, PFPeA,PFHpA PFSA: PFOS, PFHxS Emerging PFAS: 6:2 FTSA,PFBS	PFOS:0.38-2.91 ng/mL(FF), PFHxS:0.07-1.92 ng/mL (FF); PFBA: 0.02-0.09 ng/mL(SF), 6:2 FTSA:0.07-0.09 ng/mL (SF)	A reliable method for detecting exogenous chemicals in multiple biological fluids was established, revealing that couples in the preconception period are widely exposed to endocrine-disrupting chemicals, and some chemicals can enter the reproductive microenvironment, indicating potential risks to fertility.

## 6 Exposure assessment in ART studies

### 6.1 Biological matrices for PFAS measurement

In ART studies, PFAS exposure is typically assessed by measuring PFAS concentrations in serum or follicular fluid. Commonly detected PFAS in these biological matrices include PFOS, PFOA, perfluorohexane sulfonate (PFHxS), and perfluorononanoic acid (PFNA). Serum PFAS concentrations are generally considered an indicator of systemic, long-term exposure because PFAS have a long half-life in the human body. However, serum levels may not fully reflect actual exposure levels in the local ovarian microenvironment (99). Consequently, some studies have begun measuring PFAS concentrations in follicular fluid. A study by Hong et al. (2022) demonstrated a significant correlation between serum and follicular fluid PFAS levels, but noted that the ratio between the two may be influenced by protein-binding characteristics and the follicular barrier (94). Similarly, a study by Kim et al. (2020) detected multiple PFAS in the follicular fluid of infertile women and suggested that follicular fluid may more directly reflect the actual exposure environment experienced by oocytes during maturation (59). A recent paired-sample study (Marchiandi, 2024) has further shown that PFAS concentrations in blood are positively correlated with those measured in follicular fluid, supporting the use of serum as a practical indicator of internal exposure while also highlighting matrix-specific differences (100). In contrast, although PFAS have also been detected in seminal fluid, evidence regarding whether similar blood–semen correlations exist remains limited and less well characterized, indicating an important gap in male exposure assessment.

### 6.2 Temporal considerations in exposure assessment

PFAS have long half-lives in the human body; for example, the half-lives of PFOS and PFOA can span several years. Consequently, a single blood test can generally provide a good indication of long-term exposure levels. However, time-related issues may still arise in ART studies. ART treatment often involves multiple cycles, and ovarian physiology may vary between cycles. Additionally, dietary habits, lifestyle, and levels of environmental exposure may also change over time. Consequently, some studies recommend adopting multi-time-point sampling or longitudinal study designs in ART cohorts to more accurately assess the impact of PFAS exposure on ART outcomes (45).

### 6.3 Statistical approaches in ART exposure studies

In existing studies, most researchers have primarily used single-pollutant models to assess the relationship between PFAS and ART outcomes. For example, studies by Hong et al. (2022) and Shen et al. (2023) primarily used generalized linear models (GLMs) or logistic regression models to analyze the association between PFAS exposure and IVF outcomes. These models are suitable for evaluating the relationship between PFAS exposure and binary outcomes such as clinical pregnancy, the proportion of high-quality embryos, or fertilization success rates (45, 94).

In some studies, Poisson regression models have been used to analyze the relationship between PFAS exposure and oocyte maturation rates, fertilization rates, and high-quality embryo rates, as these metrics are typically expressed as count or proportion data. Compared to traditional linear models, Poisson regression is better suited for handling data with such distribution characteristics (66).

However, since humans are typically exposed to multiple PFAS simultaneously in real-world environments, an increasing number of studies in recent years have adopted mixed-pollutant analysis methods. For example, Bj¨orvang et al. (2023) employed weighted quantile sum regression (WQS) to assess the effects of multiple environmental pollutants on female fertility; this method can identify the pollutant contributing most significantly to the overall effect among highly correlated pollutants (67).

Another commonly used method is quantile g-computation (QGC), which estimates the overall effect of a mixture of pollutants on an outcome while simultaneously assessing the positive or negative contributions of individual pollutants to the overall effect (4). In contrast, Bayesian kernel machine regression (BKMR) can identify nonlinear relationships and potential interactions among pollutants, giving it a unique advantage in complex environmental exposure studies (96, 98).

Overall, with the advancement of statistical methods, ART research is gradually shifting from single-pollutant analysis to mixed-pollutant analysis, which will facilitate a more comprehensive understanding of the potential effects of PFAS exposure on reproductive outcome.

## 7 Mechanisms of PFAS effects on reproductive health

### 7.1 Endocrine-disrupting effects

**Figure 3:**
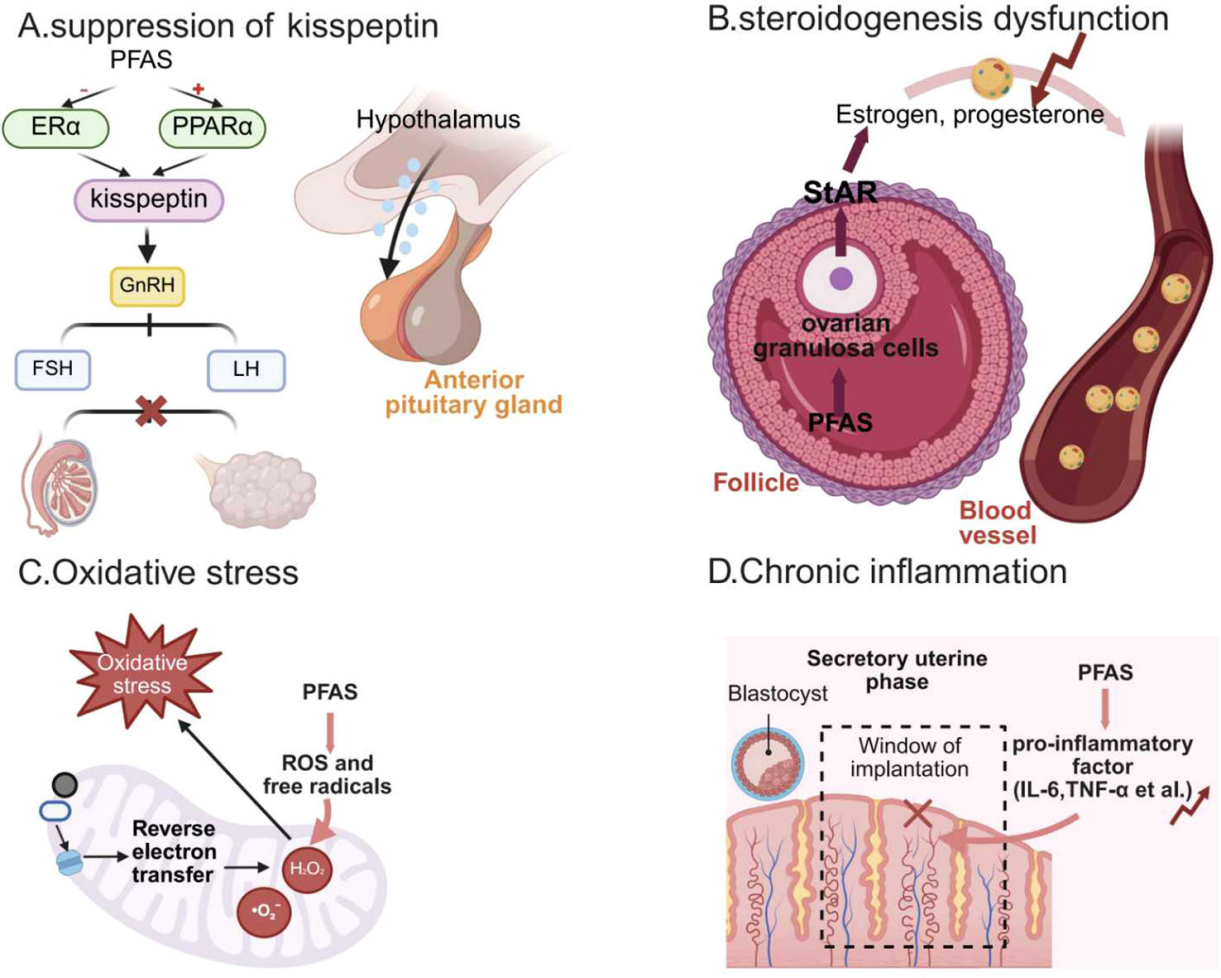
Mechanisms of PFAS-induced reproductive toxicity. **(A)** suppression of kisspeptin. PFAS can reduce hypothalamic kisspeptin expression by antagonizing ERα and activating PPARα. This suppression disrupts the subsequent GnRH–FSH/LH regulatory cascade, ultimately impairing follicular development and ovulatory function. **(B)** Steroidogenesis dysfunction. PFAS can accumulate within follicles and reduce the expression of StAR, leading to decreased secretion of key steroid hormones such as estrogen and progesterone. Impaired steroid production disrupts follicular maturation and corpus luteum function, thereby affecting the reproductive cycle and fertility. **(C)** Oxidative stress. PFAS exposure promotes mitochondrial production of ROS, including superoxide anion (O_2_^-^) and hydrogen peroxide (H_2_O_2_), and may enhance ROS generation through mechanisms such as reverse electron transport. Excessive ROS triggers oxidative stress, damaging cellular structures and disrupting ovarian and endocrine homeostasis. **(D)** Chronic inflammation. PFAS can increase the expression of pro-inflammatory factors (IL-6, TNF-α, et al.) during the uterine secretory phase, which coincides with the critical “receptive window” required for embryo implantation. An inflammatory microenvironment shortens or disrupts this implantation window, reducing the probability of successful embryo attachment and early pregnancy establishment. This figure was created by Biorender.com.

PFAS, as a class of widespread and persistent environmental pollutants, have been demonstrated to exert significant endocrine-disrupting effects. They primarily disrupt normal endocrine homeostasis by interfering with hormone synthesis, secretion, and metabolism, thereby adversely affecting reproductive health (**Figure 3**). Evidence derived from a combination of *in vivo* animal studies, *in vitro* cellular experiments, and human epidemiological observations indicates that these endocrine-disrupting effects are compound-specific rather than uniform across all PFAS.

PFAS disrupt the normal function of the hypothalamic-pituitary-gonadal axis (HPG axis). *In vivo* studies in mice have shown that specific PFAS compounds, including PFOA, PFHxS, and PFOS, can interfere with neuroendocrine regulation. PFAS may antagonize estrogen receptor alpha (ERα) or activate peroxisome proliferator-activated receptor alpha (PPARα) (e.g., PFOA-induced hepatic FGF21 synthesis demonstrated in mouse models), inhibiting kisspeptin expression in the hypothalamus and thereby reducing gonadotropin-releasing hormone (GnRH) secretion. This leads to fluctuations in luteinizing hormone (LH) and follicle-stimulating hormone (FSH) levels, ultimately disrupting follicular development and ovulation processes (101, 102). Therefore, PFAS exposure is closely associated with dysfunction of the HPG axis and alterations in hormonal homeostasis in females (103)”. Consistently, PFAS are recognized as endocrine-disrupting chemicals (EDCs) that interfere with reproductive hormone regulation, including gonadotropins and sex hormones (46).

At the level of ovarian granulosa cells, PFAS can reduce the expression of steroid synthesis acute regulatory protein (StAR), leading to decreased secretion of estrogen and androgen, which in turn affects follicular maturation and corpus luteum function. *In vitro* studies using human granulosa cells (hGCs) have demonstrated that exposure to PFAS mixtures and individual compounds, such as PFOA and PFOS, leads to downregulation of StAR expression, accompanied by altered steroid hormone synthesis, suggesting impaired granulosa cell function (104, 105).

Beyond steroidogenesis, emerging evidence indicates that PFAS can modulate intracellular signaling pathways in ovarian granulosa cells. *In vitro* experiments in human granulosa cell lines (e.g., HGrC1) have shown that PFOA exposure promotes cell proliferation and alters the expression of cell cycle–related genes, accompanied by changes in the Hippo pathway effector YAP1 (106). Consistently, ex vivo studies using cultured neonatal mouse ovaries have demonstrated that PFOA exposure can enhance follicular growth and regulate the expression of genes involved in the Hippo signaling pathway (107). These findings suggest that PFAS may influence ovarian function not only by disrupting steroidogenesis but also by altering key regulatory pathways involved in cell proliferation and tissue organization. Although bioinformatic analyzes have proposed potential links between PFAS exposure, Hippo/YAP1 signaling, and polycystic ovary syndrome (PCOS), direct mechanistic evidence supporting this connection remains limited (108).

Simultaneously, intercellular communication may represent an additional target of PFAS toxicity. Evidence from *in vitro* studies in non-reproductive cell models (e.g., osteoblasts) indicates that PFOS and PFOA can impair gap junction intercellular communication (GJIC) via connexin 43–mediated mechanisms. In addition, *in vitro* studies using human myometrial cell lines have shown that environmentally relevant concentrations of PFAS, including PFOS, PFOA, and PFAS mixtures, can alter cellular proliferation, migration, and gene transcription, accompanied by disruption of GJIC and signaling pathways related to cell survival (109, 110). Given the essential role of GJIC in coordinating communication within the cumulus cell–oocyte complex (COC), these findings raise the possibility that PFAS may interfere with cell–cell signaling in the follicular microenvironment. However, direct evidence of GJIC disruption in ovarian or COC-specific models remains limited.

Furthermore, PFAS disruption extends beyond the gonadal axis. *In vitro* studies using thyroid follicular cells have demonstrated that PFOS can inhibit iodide uptake, while other experimental systems have shown that PFAS exposure can alter thyroid peroxidase activity, thereby indirectly interfering with reproductive endocrine regulation. Epidemiological studies increasingly link PFAS exposure to thyroid dysfunction and altered pregnancy outcomes (111, 112). Overall, epidemiological and toxicological evidence indicates a significant association between PFAS exposure and menstrual cycle disorders, diminished ovarian function, and reduced fertility in women (113). These findings underscore the role of PFASs as clinically relevant endocrine-disrupting chemicals with substantial implications for reproductive health.

### 7.2 Reproductive toxicity on germ cells

The toxic effects of PFAS on germ cells represent a central mechanism underlying their adverse impact on reproductive health. Research has demonstrated that PFAS can directly damage germ cells, including impairing the quality and function of both eggs and sperm.

In males, PFAS disrupts the blood-testis barrier by interfering with steroid synthesis and the normal regulation of the HPG axis, thereby inducing spermatocyte apoptosis. This ultimately leads to reduced sperm count, decreased motility, and morphological abnormalities (44, 114). Animal studies further revealed that PFAS exposure disrupts mitotic and meiotic processes in seminiferous tubule cells, interfering with critical stages of spermatogenesis and indicating pronounced spermatogenic toxicity (115). PFAS exposure has also been associated with alterations in androgen signaling, including reduced testosterone levels and compensatory increases in gonadotropins such as FSH, which may further contribute to impaired spermatogenesis and sperm quality (54).

In females, PFAS exposure is closely associated with reduced ovarian reserve and impaired oocyte quality, manifested as delayed follicular development and accelerated reproductive aging (116, 117). Mechanistically, PFAS not only exert effects by influencing hormone synthesis within the ovaries and intercellular signaling, but typical PFAS, such as PFDA, also activate the mitochondrial-derived apoptosis pathway mediated by Caspase-9 and Caspase-3 while inhibiting Caspase-8 activity. Simultaneously, they trigger necroptosis via the RIPK1-RIPK3-MLKL axis, leading to the loss of ovarian granulosa cells and follicular atresia (118).

Additionally, PFAS induces significant oxidative stress by promoting reactive oxygen species (ROS) production and suppressing superoxide dismutase (SOD1) expression and activity, leading to decreased mitochondrial membrane potential and energy metabolism disorders. Mitochondrial dysfunction and DNA damage jointly exacerbate structural damage and apoptotic tendencies in germ cells (6, 119, 120). In male reproductive systems, increased oxidative stress in sperm has been associated with lipid peroxidation of sperm membranes, reduced motility, and impaired fertilization capacity (121). Overall, PFAS systematically impair the biological functions of oocytes and sperm through a chain reaction involving oxidative stress, mitochondrial damage, and apoptosis, thereby affecting fertility and pregnancy outcomes.

In the context of ART, these mechanistic disruptions manifest as measurable alterations in gamete quality. PFAS have been detected in follicular fluid, suggesting direct exposure of the oocyte microenvironment and a potential impact on oocyte development and competence (6, 94). Experimental studies further indicate that PFAS mixtures may impair oocyte quality and reduce oocyte yield by disrupting mitochondrial function and hormone secretion in granulosa cells (119). In addition, PFAS exposure has been associated with altered reproductive endocrine function and impaired sperm motility and morphology, providing further evidence that germ cell toxicity may translate into compromised gamete quality in ART settings (122).

### 7.3 Potential risks to pregnancy and fetal development

PFAS pose potential risks to pregnancy and fetal development. These risks have become a central focus in environmental and reproductive health research. A growing body of epidemiological and experimental evidence suggests that prenatal PFAS exposure has been associated, in some studies, with adverse pregnancy outcomes such as miscarriage, preterm birth, and reduced birth weight, as well as alterations in placental function; however, these findings are not entirely consistent across studies (46, 82, 86, 87). At the mechanistic level, PFAS can interfere with pregnancy maintenance through multiple pathways (123). First, PFAS can cross the placental barrier. Numerous human paired maternal blood-umbilical cord blood studies and placental/fetal organ testing indicate that common PFAS (such as PFOS, PFOA, etc.) can traverse the placenta and be detected in umbilical cord blood and fetal tissues. This demonstrates that fetuses are exposed to these persistent chemicals while still in the womb (124, 125). Animal (rodent) studies consistently demonstrate that exposure during pregnancy or lactation results in detectable levels of PFAS in fetal/neonatal blood and organs, accompanied by developmental and behavioral alterations. This supports maternal-fetal and lactation-infant transfer of PFAS and their associated biological consequences (126). Regarding the transport mechanism, existing evidence primarily indicates transcellular transport. Plasma protein binding and organic anion transporters (OAT/OATP/URAT) in the placental membranes jointly determine the transplacental kinetics of PFAS (127, 128); Studies directly showing paracellular pathways as the main route in placental models are limited. However, many other barrier models (epithelial/endothelial) suggest that PFAS disrupt tight junctions and increase bypass permeability. This suggests that bypass permeability may become a significant secondary mechanism under conditions of inflammation or barrier compromise (129, 130). Of note, PFAS exposure can damage mitochondrial structure and membrane potential in trophoblast cells, induce oxidative stress and apoptosis, thereby impairing placental angiogenesis and nutrient exchange functions (120). Additionally, PFAS can disrupt placental endocrine balance, alter the expression of estrogen- and progesterone-related genes, and weaken the placenta’s supportive role in maintaining pregnancy (131). Placental transfer of PFAS enables their accumulation in the fetus. Recent studies have detected multiple PFAS compounds in meconium samples, providing quantifiable biological evidence of fetal exposure (132).

Fetal exposure to PFAS is closely associated with developmental abnormalities in the nervous, immune, and endocrine systems, including neurobehavioral developmental delays and thyroid hormone disruption (117). Additionally, the epigenetic effects of PFAS are gaining increasing attention. Paternal PFAS exposure can induce DNA methylation drift, abnormal histone modifications (e.g., H3K4me3 and H3K27ac), and miRNA reprogramming in sperm. These epigenetic marks are partially retained after fertilization, driving transcriptional reprogramming of lipid metabolism and inflammatory pathways in early embryos and offspring livers (133–135). Several animal model studies have demonstrated that PFAS can cause multigenerational or transgenerational phenotypic and gene expression alterations (e.g., in zebrafish and certain rodent studies). However, the evidence for mechanisms underlying transgenerational “epigenetically stable inheritance” remains incomplete, and results vary across species and compounds. Therefore, generalizations should be avoided, but clear experimental support indicates the need for attention (136). Overall, PFAS disrupt maternal-fetal homeostasis through multiple mechanisms, including placental barrier transport, mitochondrial dysfunction, oxidative stress, and epigenetic reprogramming, thereby increasing the risk of pregnancy complications and fetal developmental abnormalities. These findings underscore the critical importance of PFAS as environmental pollutants in pregnancy health management and public health policy development.

## 8 Critical interpretation and translational challenges in PFAS and ART research

### 8.1 Translational challenges

Current evidence suggests that the effects of PFAS exposure on ART outcomes are stage-dependent and not uniformly manifested across the reproductive continuum. A growing number of cohort studies in women undergoing IVF/ICSI indicate that PFAS exposure is more consistently associated with early biological endpoints, such as reduced oocyte yield and impaired embryo quality, whereas associations with downstream clinical outcomes—including implantation and clinical pregnancy—are often weak or non-significant. Several studies have reported decreased oocyte numbers or high-quality embryo rates without corresponding reductions in implantation or pregnancy success, suggesting a potential attenuation of PFAS effects along the ART pathway (45, 58). This discrepancy likely reflects the inherently multifactorial nature of ART success, where embryo competence, endometrial receptivity, and clinical decision-making collectively determine outcomes, thereby reducing the sensitivity of distal endpoints to environmental exposures. Mechanistic studies further support biological plausibility, demonstrating that PFAS can interfere with steroidogenesis, lipid metabolism, and mitochondrial function in ovarian cells, processes essential for oocyte maturation and early embryonic development (113, 137).

Interpretation of these findings is further complicated by the translational gap between animal models and human reproductive outcomes; substantial interspecies differences limit direct extrapolation to human ART settings. PFAS toxicokinetics differ markedly, with human half-lives extending over years compared to days or weeks in rodents, resulting in fundamentally different internal exposure profiles. In addition, reproductive physiology varies considerably between species, including ovulatory patterns and endocrine regulation, which may influence susceptibility to chemical exposure. Importantly, animal models do not replicate the clinical context of ART, where controlled ovarian stimulation, *in vitro* fertilization, and embryo culture introduce additional layers of biological and procedural complexity. As a result, while animal studies provide critical mechanistic insights, human-based evidence remains essential for understanding the clinical relevance of PFAS exposure in ART populations (138, 139).

### 8.2 Limitations of current evidence

Beyond translational considerations, several methodological limitations in existing ART studies may contribute to the observed inconsistencies. A major gap in the literature is the predominant focus on female exposure, with relatively few studies incorporating paternal PFAS levels. Emerging evidence suggests that male exposure may influence reproductive outcomes by affecting sperm quality and early embryonic development. Notably, the study by Ma et al. (2021), which simultaneously assessed maternal and paternal PFAS concentrations, indicates that paternal exposure may independently contribute to variability in IVF outcomes, underscoring the need for a couple-based exposure framework (66). Furthermore, data on PFAS concentrations in seminal fluid and their relationship to ART outcomes remain extremely limited. This lack of semen-based exposure assessment restricts the ability to directly evaluate the contribution of paternal PFAS exposure to fertilization and embryo development, representing a key gap in the current literature.

In addition, many studies rely on fresh embryo transfer cycles, in which supraphysiological hormone levels induced by controlled ovarian stimulation may impair endometrial receptivity and confound exposure–outcome associations. This may partially explain why PFAS-related effects are more consistently observed in oocyte and embryo parameters than in implantation or clinical pregnancy outcomes. The multifactorial nature of ART success further complicates interpretation, as clinical endpoints are influenced by a combination of biological, technical, and clinical factors, thereby reducing the ability to detect modest environmental effects, particularly in studies with limited statistical power (10).

Another important limitation is the insufficient consideration of real-world chemical mixtures. PFAS exposure typically occurs alongside other persistent organic pollutants, and single-compound analyzes may therefore underestimate the combined effects of multiple exposures. Recent studies employing mixture modeling approaches suggest that joint exposure metrics may better capture the relationship between environmental chemicals and reproductive outcomes (96, 98). However, such approaches are not yet widely adopted. Furthermore, heterogeneity in exposure assessment—including differences in biological matrices, timing of sample collection, and the range of PFAS compounds measured—adds another layer of complexity and may contribute to inconsistent findings across studies (59, 92).

Despite growing interest in the reproductive toxicity of PFAS, the mechanistic evidence remains incomplete and, in several aspects, insufficiently resolved. Current studies largely rely on *in vitro* systems or non-reproductive cell types, with relatively limited use of physiologically relevant models that recapitulate the complexity of the reproductive system, such as the follicular microenvironment, the cumulus cell–oocyte complex, or the testicular niche. As a result, many proposed mechanisms—particularly those involving oxidative stress, mitochondrial dysfunction, and intercellular communication—are often inferred from findings in hepatic, bone, or other somatic cell models. While these studies provide valuable insights into general cellular responses to PFAS, their direct applicability to germ cells and reproductive tissues remains uncertain (140). In addition, there is a lack of integrated models that can capture the dynamic interactions among endocrine regulation, cellular signaling, and tissue-level organization within the reproductive axis. Most available evidence focuses on isolated pathways or endpoints, making it difficult to establish a coherent mechanistic framework linking molecular perturbations to clinically relevant reproductive outcomes. Furthermore, discrepancies between experimental systems, exposure conditions, and PFAS species contribute to heterogeneity across studies, limiting the comparability and translational relevance of current findings (141, 142). Collectively, these limitations highlight the need for more targeted investigations using reproductive-specific models, including advanced *in vitro* systems and well-characterized animal models, to better elucidate the mechanisms by which PFAS affect reproductive function and ART outcome.

## 9 Future research directions and public health recommendations

### 9.1 Enhance monitoring on PFAS

PFAS have drawn widespread attention due to their persistence in the environment and potential health risks. Future research should focus on enhancing PFAS monitoring and assessment to better understand their impacts on human health and ecosystems. Existing studies indicate that PFAS are associated with various health issues, including reproductive disorders and cardiovascular diseases (143). It is crucial to establish a system that specifically monitors PFAS levels in drinking water and food. Advanced analytical techniques, such as HRMS, should be employed for non-targeted analysis to identify and quantify PFAS in the environment that have not been sufficiently studied (144). In PFAS remediation, traditional water treatment technologies often struggle to remove PFAS compounds. Because ion exchange resins are costly and difficult to regenerate, highly efficient technologies are needed to meet regulatory objectives (145). The study proposes that hybrid technologies, such as combining membrane technology with advanced oxidation processes (AOPs), can enhance remediation efficiency and reduce energy consumption (146). Current attention to emerging and alternative PFAS (such as F53-B, ADONA, and GenX) remains limited. In addition to these compounds, a growing number of emerging PFAS, including C6O4, Nafion byproducts, and ester- or vinyl ether–based fluorinated substances, have been detected in environmental matrices over the past decade (147). Despite their environmental presence, these compounds remain insufficiently characterized in human biomonitoring and epidemiological studies. This gap is particularly relevant for populations residing near fluoropolymer manufacturing facilities, where exposure to such emerging PFAS may be elevated. Future epidemiological and exposure assessment studies should therefore expand target analyte panels to include these under-recognized compounds (148). Additionally, research should focus on the combined effects of PFAS, as interactions between different PFAS may exacerbate their toxicity (149). Through these efforts, a more robust scientific basis can be provided for public health policies, thereby effectively reducing PFAS-related environmental exposure risks.

### 9.2 Policy development and public awareness enhancement

To address the health risks posed by PFAS, policymakers need to take more proactive measures to restrict the use and release of these substances. Consideration should be given to implementing stricter regulations to limit PFAS in industrial and consumer products, while encouraging businesses to adopt alternative materials (150, 151). The European Chemicals Agency (ECHA) has proposed a ban on over 12,000 PFAS (152). Regarding future regulatory approaches, there is a need to transition from regulating individual compounds to conducting risk assessments and implementing controls across the entire PFAS category. Research on the toxicity and environmental behavior of novel short-chain PFAS should be intensified, and a comprehensive regulatory framework should be established to encompass prevention, detection, and remediation. Efforts should be made to promote globally harmonized PFAS emission standards and detection methods, thereby reducing pollution transfer caused by regional regulatory disparities (35). Furthermore, raising public awareness is also crucial. Through educational and awareness campaigns, the public’s understanding of PFAS can be enhanced, enabling them to recognize potential health risks and thereby prompting consumers to make more informed decisions when selecting products (34). Governments and non-governmental organizations should collaborate on community engagement initiatives to ensure public voices are heard in policy-making processes, thereby promoting more transparent and accountable policy implementation.

### 9.3 Developing safe ART

ART has become an indispensable clinical strategy for addressing infertility worldwide, contributing significantly to population health, reproductive autonomy, and the mitigation of age-related fertility decline (95, 153). With the increasing prevalence of ART, ensuring the safety and efficacy of these techniques has become particularly important. Research indicates that exposure to PFAS may negatively impact reproductive outcomes, warranting special attention during ART procedures (66). Because ART involves tightly regulated hormonal stimulation, gamete manipulation, embryo culture, and early implantation, it may be especially vulnerable to environmental contaminants such as PFAS, making the assessment of PFAS-related risks in ART settings of critical importance (154, 155). Importantly, potential PFAS exposure in ART may arise not only from environmental background exposure but also from clinical and laboratory settings. For example, PFAS have been reported in medical-grade plastics, fluoropolymer-coated tubing, and laboratory consumables, which may be used during oocyte retrieval, embryo culture, and cryopreservation procedures (150). In addition, culture media components and water purification systems may represent indirect sources of contamination, particularly if PFAS are not routinely monitored. Although direct evidence in ART laboratories remains limited, these findings from environmental and biomedical monitoring studies highlight plausible exposure pathways that warrant further investigation (137). From a clinical perspective, several precautionary strategies may be considered to minimize PFAS-related risks in ART populations. First, biomonitoring approaches, such as measuring PFAS concentrations in serum or follicular fluid, could be explored in research settings to better characterize individual exposure profiles and identify high-risk populations (156). Second, given the long biological half-lives of certain PFAS (e.g., PFOA and PFOS), preconception interventions aimed at reducing body burden—such as minimizing dietary exposure (e.g., contaminated water, food packaging) and avoiding known exposure sources—may be beneficial, although direct interventional evidence remains limited (24). Epidemiological studies have suggested that drinking water is a major contributor to PFAS exposure, supporting the rationale for targeted exposure-reduction strategies prior to ART treatment (137). At the laboratory level, optimizing material selection and quality control procedures represents another important direction. The use of PFAS-free or low-adsorption consumables, improved screening of culture media, and stricter regulation of medical device manufacturing may help reduce unintended contamination. In parallel, advances in analytical technologies, such as HRMS, could be incorporated into quality assurance workflows to detect a broader spectrum of PFAS compounds in laboratory environments and biological samples, thereby improving exposure assessment in ART settings (36, 37). Future research should focus on developing new ART methods to reduce reliance on harmful substances such as PFAS and explore how to enhance fertility rates and lower the risk of complications by optimizing medium and operational procedures (157). Research has begun to focus on the application of photo-biomodulation (PBM) therapy in male infertility (158). Additionally, quality control for ART-related medical devices should be strengthened to ensure their safety and efficacy during use (159). Collectively, elucidating the impact of PFAS on ART outcomes not only informs safer clinical practice but also provides a scientific basis for improving reproductive success while minimizing environmental health risks (160, 161). These measures can provide safer options for families seeking to conceive through ART while reducing potential health risks.

### 9.4 Future research directions

Building on current limitations, future research should prioritize the development of physiologically relevant, reproductive-specific models to better characterize PFAS effects on reproductive function. Advanced *in vitro* systems, such as three-dimensional follicle culture models, organoids, and co-culture systems incorporating granulosa cells and oocytes, may provide improved platforms for investigating the impact of PFAS on the follicular microenvironment and the COC. Similarly, the establishment of more refined animal models that mimic human exposure scenarios, including low-dose and mixture-based exposures, will be essential for improving the translational relevance of experimental findings (162, 163). In parallel, efforts should be made to integrate multiple levels of biological organization, linking molecular and cellular alterations to tissue-level changes and ultimately to reproductive outcomes. Multi-omics approaches, including transcriptomics, epigenomics, and metabolomics, combined with functional assays, may help to construct a more comprehensive mechanistic framework and identify key regulatory pathways involved in PFAS-induced reproductive toxicity.

From an epidemiological and analytical perspective, greater emphasis should be placed on integrating maternal and paternal exposures within unified analytical frameworks to enable a more comprehensive assessment of reproductive risk. In addition, the adoption of study designs that minimize hormonal and procedural confounding—such as frozen embryo transfer cycles—may provide clearer insights into the relationship between PFAS exposure and implantation outcomes (66).

Advances in statistical methodologies also offer important opportunities to address current analytical limitations. Approaches such as weighted quantile sum regression, Bayesian kernel machine regression, and quantile g-computation enable the evaluation of complex chemical mixtures and may help identify key contributors within correlated exposure profiles. Furthermore, given the widespread and simultaneous exposure to multiple PFAS compounds in real-world settings, future research should move beyond single-compound analyzes to investigate mixture effects and potential synergistic or antagonistic interactions (16).

Longitudinal and transgenerational studies are also warranted to evaluate the persistence of PFAS-induced alterations in germ cells and their potential impact on offspring health. Finally, greater integration between epidemiological studies and experimental research is needed. Strengthening the linkage between human cohort data and mechanistic findings—along with improved standardization across studies, including harmonized exposure assessment, consistent outcome definitions, and multicenter collaboration—will be critical to enhancing comparability, facilitating meta-analyzes, and improving risk assessment in the context of ART. Collectively, these efforts will help bridge current knowledge gaps and provide a more robust basis for understanding and mitigating the reproductive risks associated with PFAS exposure (164).

## 10 Conclusion

PFAS are persistent environmental pollutants. They have drawn increasing attention due to their potential adverse effects on reproductive outcomes in ART. Existing literature indicates that PFAS exposure may be closely associated with reduced fertility, adverse pregnancy outcomes, and fetal health issues. These findings highlight the need to consider environmental factors, such as PFAS exposure, when performing assisted reproductive procedures. However, current research remains limited, particularly in reconciling inconsistent findings across different studies.

Importantly, as highlighted throughout this review, the effects of PFAS appear to be more consistently observed at earlier stages of the reproductive process, including oocyte competence, follicular microenvironmental regulation, and embryo quality, whereas associations with downstream clinical outcomes such as implantation and pregnancy rates remain less consistent. This stage-specific pattern underscores the need to move beyond binary clinical endpoints and incorporate intermediate biological markers when assessing the reproductive toxicity of PFAS in ART settings.

Although multiple studies have indicated a negative correlation between PFAS exposure and fertility, the underlying biological mechanisms remain incompletely understood. Differences in sample selection, methodologies, exposure assessment, and outcome definitions across studies further complicate interpretation. In particular, mechanistic evidence is still largely derived from non-reproductive or simplified experimental systems, limiting direct translation to the complex hormonal and cellular environment of human reproduction. Therefore, future research should focus not only on elucidating specific molecular pathways—such as oxidative stress, endocrine disruption, and mitochondrial dysfunction—but also on validating these mechanisms in physiologically relevant reproductive models.

The effects of PFAS on pregnancy and fetal health also warrant further investigation. While existing studies have linked PFAS exposure to outcomes such as low birth weight and preterm birth, these associations are primarily derived from general population cohorts rather than ART-specific populations, and their applicability to assisted reproduction remains to be fully established. More longitudinal and, where possible, transgenerational studies are needed to assess the persistence and long-term consequences of PFAS exposure on offspring health. This is particularly important given the potential for PFAS to accumulate in reproductive tissues and be transferred across generations.

To address PFAS-related environmental pollution, countries should strengthen regulatory oversight and implement effective pollution control measures. Simultaneously, the public should also consider environmental factors when undergoing assisted reproductive technologies to enhance reproductive health outcomes. From a translational perspective, closer integration between epidemiological findings and mechanistic research will be critical for informing clinical guidance and risk assessment.

In summary, the impact of PFAS on assisted reproductive technologies represents a complex and multifaceted issue that spans environmental science, reproductive biology, and clinical medicine. While current evidence suggests that PFAS exposure may interfere with key stages of the reproductive process, substantial uncertainties remain regarding the magnitude, specificity, and clinical significance of these effects. Addressing these gaps will require coordinated efforts across experimental, epidemiological, and clinical domains. Through continued scientific investigation and evidence-based policy development, a more balanced approach to environmental protection and reproductive health can be achieved, ultimately providing stronger safeguards for individuals seeking fertility treatment and for future generations.

## References

[1] Waterfield G Rogers M Grandjean P Auffhammer M Sunding D . Reducing exposure to high levels of perfluorinated compounds in drinking water improves reproductive outcomes: Evidence from an intervention in minnesota. Environ Health. (2020) 19:42. doi: 10.1186/s12940-020-00591-0. PMID: 32321520 PMC7178962

[2] Casciani V Monseur B Cimadomo D Alvero R Rienzi L . Oocyte and embryo cryopreservation in assisted reproductive technology: Past achievements and current challenges. Fertil Steril. (2023) 120:506–20. doi: 10.1016/j.fertnstert.2023.06.005. PMID: 37290552

[3] Adamson GD Zegers-Hochschild F Dyer S . Global fertility care with assisted reproductive technology. Fertil Steril. (2023) 120:473–82. doi: 10.1016/j.fertnstert.2023.01.013. PMID: 36642305

[4] Yang L Liu R Li K Chen S Tan L Xu X . Associations between per- and polyfluoroalkyl substances and reproductive outcomes among women undergoing *in vitro* fertilization/intracytoplasmic sperm injection treatment. Reprod Toxicol. (2026) 139:109107. doi: 10.1016/j.reprotox.2025.109107. PMID: 41224152

[5] Voros C Athanasiou D Papapanagiotou I Mavrogianni D Varthaliti A Bananis K . Molecular shadows of per- and polyfluoroalkyl substances (PFASs): Unveiling the impact of perfluoroalkyl substances on ovarian function, polycystic ovarian syndrome (PCOS), and *in vitro* fertilization (IVF) outcomes. IJMS. (2025) 26:6604. doi: 10.3390/ijms26146604. PMID: 40724853 PMC12295940

[6] Tatarczuch A Gogola-Mruk J Kotarska K Polański Z Ptak A . Mitochondrial activity and steroid secretion in mouse ovarian granulosa cells are suppressed by a PFAS mixture. Toxicology. (2025) 512:154083. doi: 10.1016/j.tox.2025.154083. PMID: 39933620

[7] Zhang Y Mustieles V Korevaar TIM Martin L Sun Y Bibi Z . Association between per- and polyfluoroalkyl substances exposure and thyroid function biomarkers among females attending a fertility clinic. Environ pollut. (2024) 346:123513. doi: 10.1016/j.envpol.2024.123513. PMID: 38350534 PMC10950513

[8] Zhang Y Martin L Mustieles V Ghaly M Archer M Sun Y . Per- and polyfluoroalkyl substances exposure is associated with polycystic ovary syndrome risk among women attending a fertility clinic. Sci Tot Environ. (2024) 950:175313. doi: 10.1016/j.scitotenv.2024.175313. PMID: 39117221 PMC11357523

[9] Qiao R Guo F Ding H Sun D Hu Q Li Y . Association between novel per- and poly-fluoroalkyl substances and premature ovarian insufficiency: a case–control study. Hum Reprod Open. (2025) 2025:hoaf044. doi: 10.1093/hropen/hoaf044. PMID: 40740667 PMC12308182

[10] Xu J Wang Q Jiao X Kong P Chen S Yang W . Association between perfluorooctanoic acid-related poor embryo quality and metabolite alterations in human follicular fluid during IVF: A cohort study. Environ Health Perspect. (2025) 133:67017. doi: 10.1289/EHP15422. PMID: 40334213 PMC12176097

[11] Haimbaugh A Meyer DN Connell ML Blount-Pacheco J Tolofari D Gonzalez G . Environmental exposure to per- and polyfluorylalkyl substances (PFASs) and reproductive outcomes in the general population: A systematic review of epidemiological studies. IJERPH. (2024) 21:1615. doi: 10.3390/ijerph21121615. PMID: 39767456 PMC11675763

[12] Shi W Zhang Z Li M Dong H Li J . Reproductive toxicity of PFOA, PFOS and their substitutes: A review based on epidemiological and toxicological evidence. Environ Res. (2024) 250:118485. doi: 10.1016/j.envres.2024.118485. PMID: 38373549

[13] Wang Z Buser AM Cousins IT Demattio S Drost W Johansson O . A new OECD definition for per- and polyfluoroalkyl substances. Environ Sci Technol. (2021) 55:15575–8. doi: 10.1021/acs.est.1c06896. PMID: 34751569

[14] Buck RC Franklin J Berger U Conder JM Cousins IT De Voogt P . Perfluoroalkyl and polyfluoroalkyl substances in the environment: Terminology, classification, and origins. Integr Environ Assess Manag. (2011) 7:513–41. doi: 10.1002/ieam.258. PMID: 21793199 PMC3214619

[15] Feng J Soto‐Moreno EJ Prakash A Balboula AZ Qiao H . Adverse PFAS effects on mouse oocyte *in vitro* maturation are associated with carbon‐chain length and inclusion of a sulfonate group. Cell Prolif. (2023) 56:e13353. doi: 10.1111/cpr.13353. PMID: 36305033 PMC9890540

[16] Sunderland EM Hu XC Dassuncao C Tokranov AK Wagner CC Allen JG . A review of the pathways of human exposure to poly- and perfluoroalkyl substances (PFASs) and present understanding of health effects. J Expo Sci Environ Epidemiol. (2019) 29:131–47. doi: 10.1038/s41370-018-0094-1. PMID: 30470793 PMC6380916

[17] Pattarawat P Zhan T Fan Y Zhang J Yang H Zhang Y . Exposure to long- and short-chain per- and polyfluoroalkyl substances in mice and ovarian-related outcomes: An *in vivo* and *in vitro* study. Environ Health Perspect. (2025) 133:057024. doi: 10.1289/EHP14876. PMID: 40194260 PMC12120842

[18] Norén E Lindh C Glynn A Rylander L Pineda D Nielsen C . Temporal trends, 2000–2017, of perfluoroalkyl acid (PFAA) concentrations in serum of Swedish adolescents. Environ Int. (2021) 155:106716. doi: 10.1016/j.envint.2021.106716. PMID: 34144476

[19] Berhanu A Mutanda I Taolin J Qaria MA Yang B Zhu D . A review of microbial degradation of per- and polyfluoroalkyl substances (PFAS): Biotransformation routes and enzymes. Sci Tot Environ. (2023) 859:160010. doi: 10.1016/j.scitotenv.2022.160010. PMID: 36356780

[20] Wu S Yuan T Fu W Dong H Zhang Y Zhang M . Perfluorinated compound correlation between human serum and drinking water: Is drinking water a significant contributor? Sci Tot Environ. (2023) 873:162471. doi: 10.1016/j.scitotenv.2023.162471. PMID: 36842602

[21] Gomis MI Vestergren R Borg D Cousins IT . Comparing the toxic potency *in vivo* of long-chain perfluoroalkyl acids and fluorinated alternatives. Environ Int. (2018) 113:1–9. doi: 10.1016/j.envint.2018.01.011. PMID: 29421396

[22] Pelch KE Reade A Wolffe TAM Kwiatkowski CF . PFAS health effects database: Protocol for a systematic evidence map. Environ Int. (2019) 130:104851. doi: 10.1016/j.envint.2019.05.045. PMID: 31284092

[23] Dai Y He J He F Chen Z Jiang Y Geng Y . Exposure to environmentally relevant levels of GenX affects placental and offspring development in mice. Environ pollut. (2024) 363:125294. doi: 10.1016/j.envpol.2024.125294. PMID: 39532251

[24] Olsen GW Burris JM Ehresman DJ Froehlich JW Seacat AM Butenhoff JL . Half-life of serum elimination of perfluorooctanesulfonate,perfluorohexanesulfonate, and perfluorooctanoate in retired fluorochemical production workers. Environ Health Perspect. (2007) 115:1298–305. doi: 10.1289/ehp.10009. PMID: 17805419 PMC1964923

[25] Lau C Anitole K Hodes C Lai D Pfahles-Hutchens A Seed J . Perfluoroalkyl acids: A review of monitoring and toxicological findings. Toxicol Sci. (2007) 99:366–94. doi: 10.1093/toxsci/kfm128. PMID: 17519394

[26] Yates R Liu Z Gan J . Differential bioaccumulation of legacy and novel per- and polyfluoroalkyl substances (PFAS) in plants and soil biota: Implications for human exposure and risk mitigation. Environ Sci Technol. (2025) 59:25123–37. doi: 10.1021/acs.est.5c09225. PMID: 41253557

[27] Alam MS Abbasi A Chen G . Fate, distribution, and transport dynamics of per- and polyfluoroalkyl substances (PFASs) in the environment. J Environ Manag. (2024) 371:123163. doi: 10.1016/j.jenvman.2024.123163. PMID: 39515017

[28] Schymanski EL Zhang J Thiessen PA Chirsir P Kondic T Bolton EE . Per- and polyfluoroalkyl substances (PFAS) in PubChem: 7 million and growing. Environ Sci Technol. (2023) 57:16918–28. doi: 10.1021/acs.est.3c04855. PMID: 37871188 PMC10634333

[29] Cioni L Nikiforov V Coêlho ACMF Sandanger TM Herzke D . Total oxidizable precursors assay for PFAS in human serum. Environ Int. (2022) 170:107656. doi: 10.1016/j.envint.2022.107656. PMID: 36436462

[30] Kannan K Corsolini S Falandysz J Fillmann G Kumar KS Loganathan BG . Perfluorooctanesulfonate and related fluorochemicals in human blood from several countries. Environ Sci Technol. (2004) 38:4489–95. doi: 10.1021/es0493446. PMID: 15461154

[31] Dasu K Xia X Siriwardena D Klupinski TP Seay B . Concentration profiles of per- and polyfluoroalkyl substances in major sources to the environment. J Environ Manag. (2022) 301:113879. doi: 10.1016/j.jenvman.2021.113879. PMID: 34619593

[32] Thompson KA Ray H Gerrity D Quiñones O Dano E Prieur J . Sources of per- and polyfluoroalkyl substances in an arid, urban, wastewater-dominated watershed. Sci Tot Environ. (2024) 940:173361. doi: 10.1016/j.scitotenv.2024.173361. PMID: 38777060

[33] Zheng J Liu S Yang J Zheng S Sun B . Per- and polyfluoroalkyl substances (PFAS) and cancer: Detection methodologies, epidemiological insights, potential carcinogenic mechanisms, and future perspectives. Sci Tot Environ. (2024) 953:176158. doi: 10.1016/j.scitotenv.2024.176158. PMID: 39255941

[34] Lin Q Wang J Li J-J Su D-X Li M-L Wang J . determination of 17 perfluorinated/polyfluoroalkyl compounds in serum by high-throughput solid-phase extraction-ultra-high performance liquid chromatography-tandem mass spectrometry. Se Pu. (2025) 43:252–60. doi: 10.3724/SP.J.1123.2024.03007. PMID: 40045647 PMC11883548

[35] U.S. Environmental Protection Agency (EPA) . Multi-industry per- and polyfluoroalkyl substances (PFAS) study. Washington, DC, USA: U.S. Environmental Protection Agency (2021).

[36] Wang Z Yuan G Sun M Fan W Fan X Lai B . Decorative cosmetics and skin care products contribute significantly to short-chain perfluoroalkyl carboxylates exposure. J Haz Mater. (2025) 495:138846. doi: 10.1016/j.jhazmat.2025.138846. PMID: 40499412

[37] Marchiandi J Alghamdi W Dagnino S Green MP Clarke BO . Exposure to endocrine disrupting chemicals from beverage packaging materials and risk assessment for consumers. J Haz Mater. (2024) 465:133314. doi: 10.1016/j.jhazmat.2023.133314. PMID: 38147747

[38] Stroski K Sapozhnikova Y . Assessment of per- and polyfluoroalkyl substances (PFAS) in consumer food packaging. Chemosphere. (2026) 395:144824. doi: 10.1016/j.chemosphere.2026.144824. PMID: 41512439

[39] Polychronidou V Nag R . Human health risk assessment of per- and polyfluoroalkyl substances (PFAS). Sci Tot Environ. (2025) 1000:180428. doi: 10.1016/j.scitotenv.2025.180428. PMID: 40945080

[40] Zhao X Fu M Zhou S Han Y Zhang W Peng C . Targeted investigation of per- and polyfluoroalkyl substances from domestic cosmetics and personal care products in China and its implications for human exposure. Sci Tot Environ. (2024) 954:176207. doi: 10.1016/j.scitotenv.2024.176207. PMID: 39276996

[41] Cohen NJ Yao M Midya V India-Aldana S Mouzica T Andra SS . Exposure to perfluoroalkyl substances and women’s fertility outcomes in a Singaporean population-based preconception cohort. Sci Tot Environ. (2023) 873:162267. doi: 10.1016/j.scitotenv.2023.162267. PMID: 36801327 PMC10234267

[42] González-Alvarez ME Antwi-Boasiako C Keating AF . Effects of per- and polyfluoroalkylated substances on female reproduction. Toxics. (2024) 12:455. doi: 10.3390/toxics12070455. PMID: 39058107 PMC11280844

[43] Grung M Hjermann D Rundberget T Bæk K Thomsen C Knutsen HK . Low levels of per- and polyfluoroalkyl substances (PFAS) detected in drinking water in Norway, but elevated concentrations found near known sources. Sci Tot Environ. (2024) 947:174550. doi: 10.1016/j.scitotenv.2024.174550. PMID: 39004364

[44] Ajana R Rachoń D Gałęzowska G . Reproductive toxicity of per- and polyfluoroalkyl substances. Environ Toxicol Pharmacol. (2025) 117:104740. doi: 10.1016/j.etap.2025.104740. PMID: 40473150

[45] Shen J Mao Y Zhang H Lou H Zhang L Moreira JP . Exposure of women undergoing in-vitro fertilization to per-and polyfluoroalkyl substances: Evidence on negative effects on fertilization and high-quality embryos. Environ pollut. (2024) 359:124474. doi: 10.1016/j.envpol.2024.124474. PMID: 38992828

[46] Qu R Wang J Li X Zhang Y Yin T Yang P . Per- and polyfluoroalkyl substances (PFAS) affect female reproductive health: Epidemiological evidence and underlying mechanisms. Toxics. (2024) 12:678. doi: 10.3390/toxics12090678. PMID: 39330606 PMC11435644

[47] Claus Henn B Leonard ER Doherty BT Byrne S Hartmann N Ptolemy AS . Serum per- and polyfluoroalkyl substance (PFAS) levels and health-related biomarkers in a pilot study of skiers in New England. Environ Res. (2024) 263:120122. doi: 10.1016/j.envres.2024.120122. PMID: 39389198

[48] Starnes HM Green AJ Reif DM Belcher SM . An *in vitro* and machine learning framework for quantifying serum albumin binding of per- and polyfluoroalkyl substances. Toxicol Sci. (2025) 203:67–78. doi: 10.1093/toxsci/kfae124. PMID: 39298512 PMC11664106

[49] Fischer FC Ludtke S Thackray C Pickard HM Haque F Dassuncao C . Binding of per- and polyfluoroalkyl substances (PFAS) to serum proteins: Implications for toxicokinetics in humans. Environ Sci Technol. (2024) 58:1055–63. doi: 10.1021/acs.est.3c07415. PMID: 38166384 PMC11149785

[50] Luo K Huang W Zhang Q Liu X Nian M Wei M . Environmental exposure to legacy poly/perfluoroalkyl substances, emerging alternatives and isomers and semen quality in men: A mixture analysis. Sci Tot Environ. (2022) 833:155158. doi: 10.1016/j.scitotenv.2022.155158. PMID: 35421474

[51] Cui Q Pan Y Wang J Liu H Yao B Dai J . Exposure to per- and polyfluoroalkyl substances (PFASs) in serum versus semen and their association with male reproductive hormones. Environ pollut. (2020) 266:115330. doi: 10.1016/j.envpol.2020.115330. PMID: 32781340

[52] Sánchez-Resino E Marquès M Gutiérrez-Martín D Restrepo-Montes E Martínez MÁ Salas-Huetos A . Exploring the occurrence of organic contaminants in human semen through an innovative LC-HRMS-based methodology suitable for target and nontarget analysis. Environ Sci Technol. (2023) 57:19236–52. doi: 10.1021/acs.est.3c04347. PMID: 37934628 PMC10722465

[53] Pan Y Cui Q Wang J Sheng N Jing J Yao B . Profiles of emerging and legacy per-/polyfluoroalkyl substances in matched serum and semen samples: New implications for human semen quality. Environ Health Perspect. (2019) 127:127005. doi: 10.1289/EHP4431. PMID: 31841032 PMC6957285

[54] Petersen KU Hærvig KK Flachs EM Bonde JP Lindh C Hougaard KS . Per- and polyfluoroalkyl substances (PFAS) and male reproductive function in young adulthood; a cross-sectional study. Environ Res. (2022) 212:113157. doi: 10.1016/j.envres.2022.113157. PMID: 35318009

[55] Shi L Tao L Zong Y Gao C Han B Gao T . Seminal per- and polyfluoroalkyl substance exposure and sperm quality impairment: from toxic target to rescue. Environ Int. (2025) 200:109533. doi: 10.1016/j.envint.2025.109533. PMID: 40409068

[56] Wang H Wei K Wu Z Liu F Wang D Peng X . Association between per- and polyfluoroalkyl substances and semen quality. Environ Sci pollut Res. (2022) 30:27884–94. doi: 10.1007/s11356-022-24182-3. PMID: 36396760

[57] Sun F Lin Y Pan A Meng T-Q Xiong C-L Wang Y-X . Per- and polyfluoroalkyl substances in semen associated with repeated measures of semen quality in healthy adult men. Environ Sci Technol. (2025) 59:256–67. doi: 10.1021/acs.est.4c10425. PMID: 39745179

[58] Zeng X-W Bloom MS Wei F Liu L Qin J Xue L . Perfluoroalkyl acids in follicular fluid and embryo quality during IVF: A prospective IVF cohort in China. Environ Health Perspect. (2023) 131:027002. doi: 10.1289/EHP10857. PMID: 36723383 PMC9891133

[59] Kim YR White N Bräunig J Vijayasarathy S Mueller JF Knox CL . Per- and poly-fluoroalkyl substances (PFASs) in follicular fluid from women experiencing infertility in Australia. Environ Res. (2020) 190:109963. doi: 10.1016/j.envres.2020.109963. PMID: 32745751

[60] Gaillard L Barouki R Blanc E Coumoul X Andréau K . Per- and polyfluoroalkyl substances as persistent pollutants with metabolic and endocrine-disrupting impacts. Trends Endocrinol Metab. (2025) 36:249–61. doi: 10.1016/j.tem.2024.07.021. PMID: 39181731

[61] Panieri E Baralic K Djukic-Cosic D Buha Djordjevic A Saso L . PFAS molecules: A major concern for the human health and the environment. Toxics. (2022) 10:44. doi: 10.3390/toxics10020044. PMID: 35202231 PMC8878656

[62] Green MP Shearer C Patrick R Kabiri S Rivers N Nixon B . The perils of poly- and perfluorinated chemicals on the reproductive health of humans, livestock, and wildlife. Reprod Fertil Dev. (2024) 36:RD24034. doi: 10.1071/RD24034. PMID: 38744493

[63] Appel M Forsthuber M Ramos R Widhalm R Granitzer S Uhl M . The transplacental transfer efficiency of per- and polyfluoroalkyl substances (PFAS): A first meta-analysis. J Toxicol Environ Hlth Part B. (2022) 25:23–42. doi: 10.1080/10937404.2021.2009946. PMID: 34930098

[64] Steenland K Tinker S Frisbee S Ducatman A Vaccarino V . Association of perfluorooctanoic acid and perfluorooctane sulfonate with serum lipids among adults living near a chemical plant. Am J Epidemiol. (2009) 170:1268–78. doi: 10.1093/aje/kwp279. PMID: 19846564

[65] Hu XC Andrews DQ Lindstrom AB Bruton TA Schaider LA Grandjean P . Detection of poly- and perfluoroalkyl substances (PFASs) in U.S. drinking water linked to industrial sites, military fire training areas, and wastewater treatment plants. Environ Sci Technol Lett. (2016) 3:344–50. doi: 10.1021/acs.estlett.6b00260. PMID: 27752509 PMC5062567

[66] Ma X Cui L Chen L Zhang J Zhang X Kang Q . Parental plasma concentrations of perfluoroalkyl substances and *in vitro* fertilization outcomes. Environ pollut. (2021) 269:116159. doi: 10.1016/j.envpol.2020.116159. PMID: 33279270

[67] Björvang RD Hallberg I Pikki A Berglund L Pedrelli M Kiviranta H . Follicular fluid and blood levels of persistent organic pollutants and reproductive outcomes among women undergoing assisted reproductive technologies. Environ Res. (2022) 208:112626. doi: 10.1016/j.envres.2021.112626. PMID: 34973191

[68] Groisman L Berman T Quinn A Pariente G Rorman E Karakis I . Levels of PFAS concentrations in the placenta and pregnancy complications. Ecotoxicol Environ Saf. (2023) 262:115165. doi: 10.1016/j.ecoenv.2023.115165. PMID: 37348217

[69] Khan S Ouidir M Lemaitre N Jovanovic N Bayat S Lyon-Caen S . PFAS exposure during pregnancy: Implications for placental health and functioning. Environ Int. (2025) 197:109308. doi: 10.1016/j.envint.2025.109308. PMID: 39986002

[70] Preston EV Hivert M-F Fleisch AF Calafat AM Sagiv SK Perng W . Early-pregnancy plasma per- and polyfluoroalkyl substance (PFAS) concentrations and hypertensive disorders of pregnancy in the project viva cohort. Environ Int. (2022) 165:107335. doi: 10.1016/j.envint.2022.107335. PMID: 35696844 PMC9348856

[71] Chowdhury SF Prout N Rivera-Núñez Z Barrett E Brunner J Duberstein Z . PFAS alters placental arterial vasculature in term human placentae: A prospective pregnancy cohort study. Placenta. (2024) 149:54–63. doi: 10.1016/j.placenta.2024.03.002. PMID: 38518389 PMC10997442

[72] Szilagyi JT Freedman AN Kepper SL Keshava AM Bangma JT Fry RC . Per- and polyfluoroalkyl substances differentially inhibit placental trophoblast migration and invasion. Toxicological Sciences. (2020) 175(2):210–219. doi: 10.1093/toxsci/kfaa043 PMC7253207 32219433

[73] Mishra JS Bosse B Hoppe KK Malecki K Hetzel SJ Kumar S . Perfluoroalkyl substances (PFAS) exposure and preeclampsia risk: Impaired angiogenesis through suppression of VEGF signaling. Reprod Toxicol. (2025) 132:108827. doi: 10.1016/j.reprotox.2024.108827. PMID: 39732411 PMC11890960

[74] Yang Y Teng S Lin L Li W Zhu Z Chen T . Association of prenatal exposure to perfluoroalkyl and polyfluoroalkyl substances with fetal growth trajectories. Environ Res. (2025) 274:121331. doi: 10.1016/j.envres.2025.121331. PMID: 40057104

[75] Zhang Y Mustieles V Sun Q Coull B McElrath T Rifas-Shiman SL . Association of early pregnancy perfluoroalkyl and polyfluoroalkyl substance exposure with birth outcomes. JAMA Netw Open. (2023) 6:e2314934. doi: 10.1001/jamanetworkopen.2023.14934. PMID: 37256622 PMC10233420

[76] Ashrafi M Gosili R Hosseini R Arabipoor A Ahmadi J Chehrazi M . Risk of gestational diabetes mellitus in patients undergoing assisted reproductive techniques. Eur J Obstetr Gynecol Reprod Biol. (2014) 176:149–52. doi: 10.1016/j.ejogrb.2014.02.009. PMID: 24630294

[77] Mao D Ding G Wang Z Zhao J Li H Lei X . Associations of legacy perfluoroalkyl and polyfluoroalkyl substances, alternatives, and isomers with gestational diabetes mellitus and glucose homeostasis among women conceiving through assisted reproduction in Shanghai, China. Environ Sci pollut Res. (2024) 31:14088–102. doi: 10.1007/s11356-023-31605-2. PMID: 38273080

[78] Szilagyi JT Avula V Fry RC . Perfluoroalkyl substances (PFAS) and their effects on the placenta, pregnancy, and child development: A potential mechanistic role for placental peroxisome proliferator–activated receptors (PPARs). Curr Envir Health Rpt. (2020) 7:222–30. doi: 10.1007/s40572-020-00279-0. PMID: 32812200 PMC7473499

[79] Attema B Janssen AWF Rijkers D Van Schothorst EM Hooiveld GJEJ Kersten S . Exposure to low-dose perfluorooctanoic acid promotes hepatic steatosis and disrupts the hepatic transcriptome in mice. Mol Metab. (2022) 66:101602. doi: 10.1016/j.molmet.2022.101602. PMID: 36115532 PMC9526138

[80] Evans N Conley JM Cardon M Hartig P Medlock-Kakaley E Gray LE . *In vitro* activity of a panel of per- and polyfluoroalkyl substances (PFAS), fatty acids, and pharmaceuticals in peroxisome proliferator-activated receptor (PPAR) alpha, PPAR gamma, and estrogen receptor assays. Toxicol Appl Pharmacol. (2022) 449:116136. doi: 10.1016/j.taap.2022.116136. PMID: 35752307 PMC9341220

[81] Birru RL Liang H-W Farooq F Bedi M Feghali M Haggerty CL . A pathway level analysis of PFAS exposure and risk of gestational diabetes mellitus. Environ Health. (2021) 20:63. doi: 10.1186/s12940-021-00740-z. PMID: 34022907 PMC8141246

[82] Jensen TK Andersen LB Kyhl HB Nielsen F Christesen HT Grandjean P . Association between perfluorinated compound exposure and miscarriage in Danish pregnant women. PloS One. (2015) 10:e0123496. doi: 10.1371/journal.pone.0123496. PMID: 25848775 PMC4388566

[83] Park N-Y Cho SW Seo YE Chae H Lee I Lee YA . Exposure to and transplacental transfer of per- and polyfluoroalkyl substances in a twin pregnancy cohort in Korea. Environ Sci Technol. (2024) 58:21120–30. doi: 10.1021/acs.est.4c04915. PMID: 39503683

[84] Nian M Zhou W Feng Y Wang Y Chen Q Zhang J . Emerging and legacy PFAS and cytokine homeostasis in women of childbearing age. Sci Rep. (2022) 12:6517. doi: 10.1038/s41598-022-10501-8. PMID: 35444213 PMC9021217

[85] Midgett K Peden-Adams MM Gilkeson GS Kamen DL . *In vitro* evaluation of the effects of perfluorooctanesulfonic acid (PFOS) and perfluorooctanoic acid (PFOA) on IL-2 production in human T-cells. J Appl Toxicol. (2015) 35(5):459–465. doi: 10.1002/jat.3037 PMC4305032 25056757

[86] Qin X-D Zhou Y Bloom MS Qian Z( Geiger SD Vaughn MG . Prenatal exposure to PFAS, associations with preterm birth and modification by maternal estrogen levels: The Maoming Birth Study. Environ Health Perspect. (2023) 131:117006. doi: 10.1289/EHP11377. PMID: 37962440 PMC10644897

[87] Washino N Saijo Y Sasaki S Kato S Ban S Konishi K . Correlations between prenatal exposure to perfluorinated chemicals and reduced fetal growth. Environ Health Perspect. (2009) 117:660–7. doi: 10.1289/ehp.11681. PMID: 19440508 PMC2679613

[88] Davidsen N Lauvås AJ Myhre O Ropstad E Carpi D Gyves EM . Exposure to human relevant mixtures of halogenated persistent organic pollutants (POPs) alters neurodevelopmental processes in human neural stem cells undergoing differentiation. Reprod Toxicol. (2021) 100:17–34. doi: 10.1016/j.reprotox.2020.12.013. PMID: 33333158 PMC7992035

[89] Choi JW Parenti M Slupsky CM Tancredi DJ Schmidt RJ Shin H-M . Maternal serum and placental metabolomes in association with prenatal exposure to per- and polyfluoroalkyl substances and their relevance to child neurodevelopment in an ASD-enriched cohort. Environ pollut. (2025) 383:126811. doi: 10.1016/j.envpol.2025.126811. PMID: 40645267 PMC12328018

[90] Shin H-M Bennett DH Calafat AM Tancredi D Hertz-Picciotto I . Modeled prenatal exposure to per- and polyfluoroalkyl substances in association with child autism spectrum disorder: a case-control study. Environ Res. (2020) 186:109514. doi: 10.1016/j.envres.2020.109514. PMID: 32353786 PMC7363534

[91] Wang H Luo F Zhang Y Yang X Zhang S Zhang J . Prenatal exposure to perfluoroalkyl substances and child intelligence quotient: Evidence from the Shanghai birth cohort. Environ Int. (2023) 174:107912. doi: 10.1016/j.envint.2023.107912. PMID: 37023630

[92] Petro EML D’Hollander W Covaci A Bervoets L Fransen E De Neubourg D . Perfluoroalkyl acid contamination of follicular fluid and its consequence for *in vitro* oocyte developmental competence. Sci Tot Environ. (2014) 496:282–8. doi: 10.1016/j.scitotenv.2014.07.028. PMID: 25089690

[93] McCoy JA Bangma JT Reiner JL Bowden JA Schnorr J Slowey M . Associations between perfluorinated alkyl acids in blood and ovarian follicular fluid and ovarian function in women undergoing assisted reproductive treatment. Sci Tot Environ. (2017) 605–606:9–17. doi: 10.1016/j.scitotenv.2017.06.137. PMID: 28651210 PMC11276832

[94] Hong A Zhuang L Cui W Lu Q Yang P Su S . Per- and polyfluoroalkyl substances (PFAS) exposure in women seeking *in vitro* fertilization-embryo transfer treatment (IVF-ET) in China: Blood-follicular transfer and associations with IVF-ET outcomes. Sci Tot Environ. (2022) 838:156323. doi: 10.1016/j.scitotenv.2022.156323. PMID: 35636536

[95] Boney SS Christen C Wetmore BA Murr AS Raburn D Young SL . First evidence of legacy and emerging per- and polyfluoroalkyl substances (PFAS) in the follicular fluid of a cohort of North Carolina *in vitro* fertilization (IVF) patients. Reprod Toxicol. (2026) 139:109102. doi: 10.1016/j.reprotox.2025.109102. PMID: 41197966 PMC13487813

[96] Bellavia A Zou R Björvang RD Roos K Sjunnesson Y Hallberg I . Association between chemical mixtures and female fertility in women undergoing assisted reproduction in Sweden and Estonia. Environ Res. (2023) 216:114447. doi: 10.1016/j.envres.2022.114447. PMID: 36181890 PMC9729501

[97] Malhotra N Mahey R Singh N Kriplani A . Ovarian sensitivity index (OSI): Validating the use of a marker for ovarian responsiveness in IVF. J Reprod Infertil. (2019) 20(2):83–88. PMC6486569 31058052

[98] Lefebvre T Fréour T Duval G Ploteau S Marchand P Le Bizec B . Associations between internal concentrations of fluorinated and organochlorinated chemicals in women and *in vitro* fertilization outcomes: A multi-pollutant study. Environ pollut. (2022) 313:120087. doi: 10.1016/j.envpol.2022.120087. PMID: 36087895

[99] Kang Q Gao F Zhang X Wang L Liu J Fu M . Nontargeted identification of per- and polyfluoroalkyl substances in human follicular fluid and their blood-follicle transfer. Environ Int. (2020) 139:105686. doi: 10.1016/j.envint.2020.105686. PMID: 32278886

[100] Marchiandi J Dagnino S Zander-Fox D Green MP Clarke BO . Characterization of chemical exposome in a paired human preconception pilot study. Environ Sci Technol. (2024) 58:20352–65. doi: 10.1021/acs.est.4c04356. PMID: 39508786

[101] Zhang Y Cao X Chen L Qin Y Xu Y Tian Y . Exposure of female mice to perfluorooctanoic acid suppresses hypothalamic kisspeptin‐reproductive endocrine system through enhanced hepatic fibroblast growth factor 21 synthesis, leading to ovulation failure and prolonged dioestrus. J Neuroendocrinol. (2020) 32:e12848. doi: 10.1111/jne.12848. PMID: 32307816

[102] Yin X Di T Cao X Liu Z Xie J Zhang S . Chronic exposure to perfluorohexane sulfonate leads to a reproduction deficit by suppressing hypothalamic kisspeptin expression in mice. J Ovarian Res. (2021) 14:141. doi: 10.1186/s13048-021-00903-z. PMID: 34706750 PMC8555149

[103] Wang X Bai Y Tang C Cao X Chang F Chen L . Impact of perfluorooctane sulfonate on reproductive ability of female mice through suppression of estrogen receptor α-activated kisspeptin neurons. Toxicol Sci. (2018) 165:475–86. doi: 10.1093/toxsci/kfy167. PMID: 29939337

[104] Feng X Wang X Cao X Xia Y Zhou R Chen L . Chronic exposure of female mice to an environmental level of perfluorooctane sulfonate suppresses estrogen synthesis through reduced histone H3K14 acetylation of the StAR promoter leading to deficits in follicular development and ovulation. Toxicol Sci. (2015) 148:368–79. doi: 10.1093/toxsci/kfv197. PMID: 26358002

[105] Clark KL Shukla M George JW Gustin S Rowley MJ Davis JS . An environmentally relevant mixture of per- and polyfluoroalkyl substances (PFAS) impacts proliferation, steroid hormone synthesis, and gene transcription in primary human granulosa cells. Toxicol Sci. (2024) 200:57–69. doi: 10.1093/toxsci/kfae049. PMID: 38603627 PMC11199914

[106] Clark KL George JW Hua G Davis JS . Perfluorooctanoic acid promotes proliferation of the human granulosa cell line HGrC1 and alters expression of cell cycle genes and Hippo pathway effector YAP1. Reprod Toxicol. (2022) 110:49–59. doi: 10.1016/j.reprotox.2022.03.011. PMID: 35346789 PMC10364788

[107] Clark KL Davis JS . Perfluorooctanoic acid (PFOA) promotes follicular growth and alters expression of genes that regulate the cell cycle and the Hippo pathway in cultured neonatal mouse ovaries. Toxicol Appl Pharmacol. (2022) 454:116253. doi: 10.1016/j.taap.2022.116253. PMID: 36152675 PMC10416762

[108] Xu X Zhang X Chen J Du X Sun Y Zhan L . Exploring the molecular mechanisms by which per- and polyfluoroalkyl substances induce polycystic ovary syndrome through in silico toxicogenomic data mining. Ecotoxicol Environ Saf. (2024) 275:116251. doi: 10.1016/j.ecoenv.2024.116251. PMID: 38537477

[109] Yang S Chen M Yang D Deng F Guo X . Perfluorooctanoic acid and perfluorooctane sulfonate inhibit *in vitro* osteogenesis: possible role of connexin 43-mediated gap-junctional intercellular communication. Arch Toxicol. (2025) 99:2565–76. doi: 10.1007/s00204-025-04019-x. PMID: 40100396

[110] Clark KL George JW . Environmentally relevant concentrations of individual per- and polyfluoroalkyl substances (PFAS) and a PFAS mixture impact proliferation, migration, and gene transcription in a human myometrial cell line. Toxicology. (2025) 515:154173. doi: 10.1016/j.tox.2025.154173. PMID: 40334771 PMC12424527

[111] Song M Kim Y-J Park Y-K Ryu J-C . Changes in thyroid peroxidase activity in response to various chemicals. J Environ Monit. (2012) 14:2121. doi: 10.1039/c2em30106g. PMID: 22699773

[112] Conti A Strazzeri C Rhoden KJ . Perfluorooctane sulfonic acid, a persistent organic pollutant, inhibits iodide accumulation by thyroid follicular cells *in vitro*. Mol Cell Endocrinol. (2020) 515:110922. doi: 10.1016/j.mce.2020.110922. PMID: 32621861

[113] Rickard BP Rizvi I Fenton SE . Per- and poly-fluoroalkyl substances (PFAS) and female reproductive outcomes: PFAS elimination, endocrine-mediated effects, and disease. Toxicology. (2022) 465:153031. doi: 10.1016/j.tox.2021.153031. PMID: 34774661 PMC8743032

[114] Gasparini C Iori S Pietropoli E Bonato M Giantin M Barbarossa A . Sub-acute exposure of male guppies (poecilia reticulata) to environmentally relevant concentrations of PFOA and GenX induces significant changes in the testis transcriptome and reproductive traits. Environ Int. (2024) 187:108703. doi: 10.1016/j.envint.2024.108703. PMID: 38705092

[115] Eggert A Cisneros-Montalvo S Anandan S Musilli S Stukenborg J-B Adamsson A . The effects of perfluorooctanoic acid (PFOA) on fetal and adult rat testis. Reprod Toxicol. (2019) 90:68–76. doi: 10.1016/j.reprotox.2019.08.005. PMID: 31412280

[116] Wang W Hong X Zhao F Wu J Wang B . The effects of perfluoroalkyl and polyfluoroalkyl substances on female fertility: A systematic review and meta-analysis. Environ Res. (2023) 216:114718. doi: 10.1016/j.envres.2022.114718. PMID: 36334833

[117] Wu J Harlow SD Randolph JF Gold EB Park SK . Endocrine-disrupting chemicals and female reproductive aging. Semin Reprod Med. (2024) 42:330–60. doi: 10.1055/s-0044-1801388. PMID: 39879998

[118] Liu Z Cui Z Li C Lu K Chen K Cui W . Exposure to perfluorodecanoic acid impairs follicular development via inducing granulosa cell necroptosis. Ecotoxicol Environ Saf. (2024) 287:117268. doi: 10.1016/j.ecoenv.2024.117268. PMID: 39547057

[119] Jiao X Liu N Xu Y Qiao H . Perfluorononanoic acid impedes mouse oocyte maturation by inducing mitochondrial dysfunction and oxidative stress. Reprod Toxicol. (2021) 104:58–67. doi: 10.1016/j.reprotox.2021.07.002. PMID: 34246765 PMC8477654

[120] Hofmann A Mishra JS Yadav P Dangudubiyyam SV Blesson CS Kumar S . PFOS impairs mitochondrial biogenesis and dynamics and reduces oxygen consumption in human trophoblasts. J Environ Sci Public Health. (2023) 07(4):164–175. doi: 10.26502/jesph.96120197 PMC10621633 37920428

[121] Marinaro C Bianchi AR Guerretti V Barricelli G Berman B Bertola F . Molecular alterations in semen of per-and polyfluoroalkyl substance exposed subjects: Association between DNA integrity, antioxidant capacity and lipoperoxides. Antioxidants. (2025) 14:792. doi: 10.3390/antiox14070792. PMID: 40722896 PMC12291827

[122] Lockington C Favetta LA . How per- and poly-fluoroalkyl substances affect gamete viability and fertilization capability: Insights from the literature. JoX. (2024) 14:651–78. doi: 10.3390/jox14020038. PMID: 38804291 PMC11130945

[123] Blake BE Cope HA Hall SM Keys RD Mahler BW McCord J . Evaluation of maternal, embryo, and placental effects in CD-1 mice following gestational exposure to perfluorooctanoic acid (PFOA) or hexafluoropropylene oxide dimer acid (HFPO-DA or GenX). Environ Health Perspect. (2020) 128:027006. doi: 10.1289/EHP6233. PMID: 32074459 PMC7064328

[124] Mamsen LS Björvang RD Mucs D Vinnars M-T Papadogiannakis N Lindh CH . Concentrations of perfluoroalkyl substances (PFASs) in human embryonic and fetal organs from first, second, and third trimester pregnancies. Environ Int. (2019) 124:482–92. doi: 10.1016/j.envint.2019.01.010. PMID: 30684806

[125] Cai D Li Q-Q Chu C Wang S-Z Tang Y-T Appleton AA . High trans-placental transfer of perfluoroalkyl substances alternatives in the matched maternal-cord blood serum: Evidence from a birth cohort study. Sci Tot Environ. (2020) 705:135885. doi: 10.1016/j.scitotenv.2019.135885. PMID: 31841927

[126] Kaye E Marques E Agudelo Areiza J Modaresi SMS Slitt A . Exposure to a PFOA, PFOS and PFHxS mixture during gestation and lactation alters the liver proteome in offspring of CD-1 mice. Toxics. (2024) 12:348. doi: 10.3390/toxics12050348. PMID: 38787127 PMC11126053

[127] Xu Y Sui X Li J Zhang L Wang P Liu Y . Early-life exposure to per- and polyfluoroalkyl substances: Analysis of levels, health risk and binding abilities to transport proteins. Eco-Environm Health. (2024) 3:308–16. doi: 10.1016/j.eehl.2024.04.007. PMID: 39258237 PMC11385757

[128] Chen H Wei S Li J Zhong Z Chen D . Transplacental transport of per- and polyfluoroalkyl substances (PFAS): Mechanism exploration via BeWo cell monolayer model. J Haz Mater. (2024) 466:133205. doi: 10.1016/j.jhazmat.2023.133205. PMID: 38278074

[129] Lucas JH Wang Q Rahman I . Perfluorooctane sulfonic acid disrupts protective tight junction proteins via protein kinase D in airway epithelial cells. Toxicol Sci. (2022) 190:215–26. doi: 10.1093/toxsci/kfac096. PMID: 36106993 PMC9960012

[130] Liu QS Hao F Sun Z Long Y Zhou Q Jiang G . Perfluorohexadecanoic acid increases paracellular permeability in endothelial cells through the activation of plasma kallikrein-kinin system. Chemosphere. (2018) 190:191–200. doi: 10.1016/j.chemosphere.2017.10.002. PMID: 28987408

[131] Li Y Lu X Yu N Li A Zhuang T Du L . Exposure to legacy and novel perfluoroalkyl substance disturbs the metabolic homeostasis in pregnant women and fetuses: A metabolome-wide association study. Environ Int. (2021) 156:106627. doi: 10.1016/j.envint.2021.106627. PMID: 33991873

[132] Li J Ma D Qian C Guo B Guan R Liu C . Assessment of fetal exposure and elimination of perfluoroalkyl and polyfluoroalkyl substances: New evidence from paired serum, placenta, and meconium samples. Environ Sci Technol. (2024) 58:2260–70. doi: 10.1021/acs.est.3c08498. PMID: 38252093

[133] Tsai W-J Hsieh W-S Chen P-C Liu C-Y . Prenatal perfluoroalkyl substance exposure in association with global histone post-translational methylation in 2-year-old children. Toxics. (2024) 12:876. doi: 10.3390/toxics12120876. PMID: 39771091 PMC11679469

[134] Maxwell DL Oluwayiose OA Houle E Roth K Nowak K Sawant S . Mixtures of per- and polyfluoroalkyl substances (PFAS) alter sperm methylation and long-term reprogramming of offspring liver and fat transcriptome. Environ Int. (2024) 186:108577. doi: 10.1016/j.envint.2024.108577. PMID: 38521043

[135] Ho TC Wan HT Lee WK Lam TKY Lin X Chan TF . Effects of in utero PFOS exposure on epigenetics and metabolism in mouse fetal livers. Environ Sci Technol. (2023) 57:14892–903. doi: 10.1021/acs.est.3c05207. PMID: 37759171 PMC10569047

[136] Haimbaugh A Wu C-C Akemann C Meyer DN Connell M Abdi M . Multi- and transgenerational effects of developmental exposure to environmental levels of PFAS and PFAS mixture in zebrafish (danio rerio). Toxics. (2022) 10:334. doi: 10.3390/toxics10060334. PMID: 35736942 PMC9228135

[137] Li X Hou M Zhang F Ji Z Cai Y Shi Y . Per- and polyfluoroalkyl substances and female health concern: Gender-based accumulation differences, adverse outcomes, and mechanisms. Environ Sci Technol. (2025) 59:1469–86. doi: 10.1021/acs.est.4c08701. PMID: 39803974

[138] Vandenberg LN Colborn T Hayes TB Heindel JJ Jacobs DR Lee D-H . Hormones and endocrine-disrupting chemicals: Low-dose effects and nonmonotonic dose responses. Endocr Rev. (2012) 33:378–455. doi: 10.1210/er.2011-1050. PMID: 22419778 PMC3365860

[139] Gore AC Chappell VA Fenton SE Flaws JA Nadal A Prins GS . EDC-2: the endocrine society’s second scientific statement on endocrine-disrupting chemicals. Endocr Rev. (2015) 36:E1–E150. doi: 10.1210/er.2015-1010. PMID: 26544531 PMC4702494

[140] Blake BE Fenton SE . Early life exposure to per- and polyfluoroalkyl substances (PFAS) and latent health outcomes: A review including the placenta as a target tissue and possible driver of peri- and postnatal effects. Toxicology. (2020) 443:152565. doi: 10.1016/j.tox.2020.152565. PMID: 32861749 PMC7530144

[141] Hoadley L Watters M Rogers R Siegmann Werner L Markiewicz KV Forrester T . Public health evaluation of PFAS exposures and breastfeeding: a systematic literature review. Toxicol Sci. (2023) 194:121–37. doi: 10.1093/toxsci/kfad053. PMID: 37228093 PMC10527886

[142] Erinc A Davis MB Padmanabhan V Langen E Goodrich JM . Considering environmental exposures to per- and polyfluoroalkyl substances (PFAS) as risk factors for hypertensive disorders of pregnancy. Environ Res. (2021) 197:111113. doi: 10.1016/j.envres.2021.111113. PMID: 33823190 PMC8187287

[143] Lohmann R Abass K Bonefeld-Jørgensen EC Bossi R Dietz R Ferguson S . Cross-cutting studies of per- and polyfluorinated alkyl substances (PFAS) in arctic wildlife and humans. Sci Tot Environ. (2024) 954:176274. doi: 10.1016/j.scitotenv.2024.176274. PMID: 39304148 PMC11567803

[144] Megson D Niepsch D Spencer J Santos C Florance H MacLeod CL . Non-targeted analysis reveals hundreds of per- and polyfluoroalkyl substances (PFAS) in UK freshwater in the vicinity of a fluorochemical plant. Chemosphere. (2024) 367:143645. doi: 10.1016/j.chemosphere.2024.143645. PMID: 39476983

[145] Park M Daniels KD Wu S Ziska AD Snyder SA . Magnetic ion-exchange (MIEX) resin for perfluorinated alkylsubstance (PFAS) removal in groundwater: Roles of atomic charges for adsorption. Water Res. (2020) 181:115897. doi: 10.1016/j.watres.2020.115897. PMID: 32450335

[146] Bell EM De Guise S McCutcheon JR Lei Y Levin M Li B . Exposure, health effects, sensing, and remediation of the emerging PFAS contaminants – Scientific challenges and potential research directions. Sci Tot Environ. (2021) 780:146399. doi: 10.1016/j.scitotenv.2021.146399. PMID: 33770593

[147] Wang Z DeWitt JC Higgins CP Cousins IT . A never-ending story of per- and polyfluoroalkyl substances (PFASs)? Environ Sci Technol. (2017) 51:2508–18. doi: 10.1021/acs.est.6b04806. PMID: 28224793

[148] Li L Guo Y Ma S Wen H Li Y Qiao J . Association between exposure to per- and perfluoroalkyl substances (PFAS) and reproductive hormones in human: A systematic review and meta-analysis. Environ Res. (2024) 241:117553. doi: 10.1016/j.envres.2023.117553. PMID: 37931739

[149] Leemans M Spirhanzlova P Couderq S Le Mével S Grimaldi A Duvernois-Berthet E . A mixture of chemicals found in human amniotic fluid disrupts brain gene expression and behavior in xenopus laevis. Int J Mol Sci. (2023) 24:2588. doi: 10.3390/ijms24032588. PMID: 36768911 PMC9916464

[150] Figuière R Miaz LT Savvidou E Cousins IT . An overview of potential alternatives for the multiple uses of per- and polyfluoroalkyl substances. Environ Sci Technol. (2025) 59:2031–42. doi: 10.1021/acs.est.4c09088. PMID: 39851256 PMC11800378

[151] Yu R-S Yu H-C Yang Y-F Singh S . A global overview of per- and polyfluoroalkyl substance regulatory strategies and their environmental impact. Toxics. (2025) 13:251. doi: 10.3390/toxics13040251. PMID: 40278567 PMC12030800

[152] Spyrakis F Dragani TA . The EU’s per- and polyfluoroalkyl substances (PFAS) ban: A case of policy over science. Toxics. (2023) 11:721. doi: 10.3390/toxics11090721. PMID: 37755732 PMC10536631

[153] Calvert L Martin JH Anderson AL Bernstein IR Burke ND De Iuliis GN . Assessment of the impact of direct *in vitro* PFAS treatment on mouse spermatozoa. Reprod Fertil. (2024) 5:e230087. doi: 10.1530/RAF-23-0087. PMID: 38367345 PMC10959046

[154] Marlatt VL Bayen S Castaneda-Cortès D Delbès G Grigorova P Langlois VS . Impacts of endocrine disrupting chemicals on reproduction in wildlife and humans. Environ Res. (2022) 208:112584. doi: 10.1016/j.envres.2021.112584. PMID: 34951986

[155] Cabry R Merviel P Madkour A Lefranc E Scheffler F Desailloud R . The impact of endocrine disruptor chemicals on oocyte/embryo and clinical outcomes in IVF. Endocr Conn. (2020) 9:R134–42. doi: 10.1530/EC-20-0135. PMID: 32380469 PMC7354731

[156] Björvang RD Damdimopoulou P . Persistent environmental endocrine-disrupting chemicals in ovarian follicular fluid and *in vitro* fertilization treatment outcome in women. Upsala J Med Sci. (2020) 125:85–94. doi: 10.1080/03009734.2020.1727073. PMID: 32093529 PMC7721012

[157] Xue Y Xiong Y Cheng X Li K . Applications of laser technology in the manipulation of human spermatozoa. Reprod Biol Endocrinol. (2023) 21:93. doi: 10.1186/s12958-023-01148-9. PMID: 37865766 PMC10589983

[158] Zupin L Pascolo L Luppi S Ottaviani G Crovella S Ricci G . Photobiomodulation therapy for male infertility. Laser Med Sci. (2020) 35:1671–80. doi: 10.1007/s10103-020-03042-x. PMID: 32483749

[159] Briassoulis G Ilia S Briassouli E . Exposure to per- and polyfluoroalkyl substances (PFASs) in healthcare: Environmental and clinical insights. Life. (2025) 15:1057. doi: 10.3390/life15071057. PMID: 40724559 PMC12298592

[160] Ahmadi H Aghebati-Maleki L Rashidiani S Csabai T Nnaemeka OB Szekeres-Bartho J . Long-term effects of ART on the health of the offspring. IJMS. (2023) 24:13564. doi: 10.3390/ijms241713564. PMID: 37686370 PMC10487905

[161] Knox B Güil-Oumrait N Basagaña X Cserbik D Dadvand P Foraster M . Prenatal exposure to per- and polyfluoroalkyl substances, fetoplacental hemodynamics, and fetal growth. Environ Int. (2024) 193:109090. doi: 10.1016/j.envint.2024.109090. PMID: 39454342

[162] Shea LD Woodruff TK Shikanov A . Bioengineering the ovarian follicle microenvironment. Annu Rev BioMed Eng. (2014) 16:29–52. doi: 10.1146/annurev-bioeng-071813-105131. PMID: 24849592 PMC4231138

[163] Xiao S Zhang J Romero MM Smith KN Shea LD Woodruff TK . *In vitro* follicle growth supports human oocyte meiotic maturation. Sci Rep. (2015) 5:17323. doi: 10.1038/srep17323. PMID: 26612176 PMC4661442

[164] Braun JM . Early-life exposure to EDCs: role in childhood obesity and neurodevelopment. Nat Rev Endocrinol. (2017) 13:161–73. doi: 10.1038/nrendo.2016.186. PMID: 27857130 PMC5322271

